# Pharmaceutical Machine Learning: Virtual High-Throughput Screens Identifying Promising and Economical Small Molecule Inhibitors of Complement Factor C1s

**DOI:** 10.3390/biom8020024

**Published:** 2018-05-07

**Authors:** Jonathan J. Chen, Lyndsey N. Schmucker, Donald P. Visco

**Affiliations:** 1 Department of Biology, The University of Akron, 302 Buchtel Common, Akron, OH 44325, USA; jjc106@zips.uakron.edu; 2 Department of Chemical and Biomolecular Engineering, The University of Akron, 302 Buchtel Common, Akron, OH 44325, USA; lns41@zips.uakron.edu

**Keywords:** human complement factor C1, virtual high-throughput screening, data-mining, quantitative structure-activity relationship, drug discovery, Signature

## Abstract

When excessively activated, C1 is insufficiently regulated, which results in tissue damage. Such tissue damage causes the complement system to become further activated to remove the resulting tissue damage, and a vicious cycle of activation/tissue damage occurs. Current Food and Drug Administration approved treatments include supplemental recombinant C1 inhibitor, but these are extremely costly and a more economical solution is desired. In our work, we have utilized an existing data set of 136 compounds that have been previously tested for activity against C1. Using these compounds and the activity data, we have created models using principal component analysis, genetic algorithm, and support vector machine approaches to characterize activity. The models were then utilized to virtually screen the 72 million compound PubChem repository. This first round of virtual high-throughput screening identified many economical and promising inhibitor candidates, a subset of which was tested to validate their biological activity. These results were used to retrain the models and rescreen PubChem in a second round vHTS. Hit rates for the first round vHTS were 57%, while hit rates for the second round vHTS were 50%. Additional structure–property analysis was performed on the active and inactive compounds to identify interesting scaffolds for further investigation.

## 1. Introduction

Complement factor C1s (EC 3.4.21.42) is a subcomponent of the C1 complex in the complement system of the innate portion of the immune system [1]. The complex circulates the body at a concentration, relative to serum, of 0.17 μM [1] and is mainly produced and/or assembled in monocytes and macrophages [2,3]. Constituent parts, which are described later, can be produced by other tissues and cells as well [4]. The complement factor 1 gene for the entire complex is found on the fourth chromosome [5] and encodes for a 750 kDa pentameric glycoprotein comprised of three subcomponent proteins: one C1q, two C1r, and two C1s [1]. C1q is the pattern recognition subunit and binds to a wide variety of targets [6]. In the classical pathway, C1q recognition and binding cleaves and activates C1r, which, in turn, cleaves and activates C1s [7]. C1s then cleaves and activates complement factors 2 and 4, activating them to form C3 convertase. The convertase activates other immune responses including increased pro-inflammatory molecule signals [8,9], recruitment of macrophages [8,9], and creation of the membrane-attack complex [10].

C1, as the initiator of the classical pathway in the complement component of the immune system, is an important molecule to regulate. Failure to do so may result in the excessive activation of the classical pathway. Normally, the body produces C1 inhibitor to control C1 activation [11] by irreversibly binding and removing C1r and C1s during the complex assembly process [12,13]. Since C1 inhibitor is the only endogenous regulator of C1 [14,15], mutations to it and/or changes to physiological circumstances may lead to excessive C1 activation and deficient regulation of the classical pathway of the complement system.

In an example of excessive activation, incomplete extracellular debris removal (in the case of age-related macular degeneration [16]) or amyloid fibrils (in the case of Alzheimer’s disease [17]) would promote C1 activation and increase pro-inflammatory molecule signals [16]. Under normal circumstances, the increased activity would result in the removal of the targeted substance and protect local tissue. However, if the debris or fibrils cannot be removed, C1 activation would be promoted continuously leading to chronic inflammation and tissue damage. In an example of deficient regulation by the C1 inhibitor, hereditary angioedema is a genetic disorder due to insufficient levels or deficient activity of C1 inhibitors. Unregulated C1 activation results in inflammation and edema among other symptoms [18]. It should be noted the described examples are ones in which C1 and its subcomponents are directly involved. Dis-regulation of other complement factors and their corresponding regulators can indirectly involve and implicate C1 in other diseases [19].

In either C1 dysregulation example, treatment could involve a supplementary C1 inhibitor. This is an area of active research and drug development. By 2011, two acute C1 inhibitor supplementary treatments for hereditary angioedema were derived from donor plasma (Berinert^®^ [20,21] and Cinryze^™^ [22]). Meanwhile, a recombinant version of C1 inhibitor was produced in transgenic rabbit mammary glands (Ruconest^®^ [20,23,24]). Although less costly than emergency treatment [25,26], cost analysis information indicates that all three approved treatments still constitute a major financial burden for patients [20,22,24].

In light of the financial burden that current treatments present, cheaper alternatives are desired. All marketed therapeutics supplement either C1 inhibitor levels or activity in the patient. Another course of treatment could be inhibiting the activation of C1 [27]. Looking at the constituent composition of C1, C1s is the most desirable target: it initiates the classical pathway, is specific to the classical pathway and degrading C1q’s pattern recognition ability is undesirable. A patented antibody treatment targeting C1s was in phase 1 trials [28,29]. Besides plasma donated inhibitors or antibodies, small molecules are also another way to target and inhibit C1s. Potential small molecule inhibitors have been found [27,30,31], modified [32,33] and PEGylated [34] to circumvent shortcomings.

One approach under-utilized in C1s inhibitor discovery is computational methods. Computers have been growing in power and utility at an exponential rate and can now start to complement traditional design/search methodologies for new drug candidates [35]. In fact, computational approaches were used previously to identify how small molecules docked into C1s [31,32,33,34] but not to identify new leads. Researchers have used virtual high-throughput screening to identify leads for other factors in the complement system [36]. Success in these efforts supports the notion that such an approach can be applied to C1s as well. In this work, small molecule C1s inhibitor leads are identified in a virtual high-throughput screen (vHTS) using the computational technology available today.

One important aspect to consider is the number of molecules that need to be examined as a potential candidate. The number of possible molecules grows exponentially with the inclusion of different factors (e.g., types and numbers of different atoms and bonds, branching and cyclization, etc.) and “grows steeply”with increasing molecular weight [37]. Even a narrow subset can be extremely large. For example, a conservative estimate for the number of possible 30-atom molecules containing only C, N, O, and S atoms is $10^{60}$ [37]. This estimate does include structures yet to be synthesized but even if it is instead taken as an estimate of all candidates under consideration, $10^{60}$ compounds are still many orders of magnitudes larger than what can feasibly be tested in a reasonable amount of time for a single target. Thus, ways to identify likely active candidates and exclude likely inactive candidates are needed and necessary.

Utilizing expert knowledge, candidates likely to be inactive can be removed to “focus” candidate libraries, but the effect is limited [38]. Nevertheless, the focused libraries enable manageable, systematic testing of candidates. To increase throughput and efficiency, high-throughput screening (HTS) was developed to simultaneously test multiple candidates while also lowering the amounts of reagents used per test [39,40,41]. HTS allowed the systematic exploration of candidate pool subsets, known as compound libraries. Unfortunately, most candidates are inactive and if every compound is tested, near all resources are spent testing inactive candidates, resulting in low “hit-rates” [38]. Ways to include/exclude candidates will seemingly always be an active area of research.

A serendipitous result of HTS is the creation of large amounts of experimental data, which can be used with available characterization data from other sources (e.g., PubChem [42,43], CHEMBL [44] and ZINC15 [45]), itself a product of “big data” [46], to extend its utility in new and different ways. Mathematical models correlating structure with function can be developed and used for the purposes of focusing a candidate library *virtually* via vHTS. This way, computational techniques can complement expert knowledge to further remove likely inactive candidates and increase screening hit-rates. Thus, prior data and efforts can guide future work and increase the discovery rates for new/novel inhibitors.

One of the two major branches of vHTS approaches is molecular simulation (e.g., AutoDock, DOCK, Flex, AMBER, GROMACS, CHARMM) [47,48,49,50,51,52,53,54,55,56,57,58]. Molecular simulation uses fundamentals in mathematics, physics and chemistry to predict optimized ligand–substrate configurations. Although it requires minimal experimental data, it requires much more computationally and confidence depends on the convergence of multiple simulations. The other major branch of vHTS approaches is ligand-based scoring [55,59,60,61]. Ligand-based scoring uses data of known ligands, usually experimental results and structural data, to find other possible ligands. Although computationally cheaper, it does require enough experimental data to make accurate predictions. Additionally, predictions are usually similar to known ligands due to an inverse relationship between prediction power and extrapolation. A minor branch of vHTS is hybrid/mixed approaches to compensate for the deficiencies of either approach, albeit imperfectly [62].

**Figure 1:**
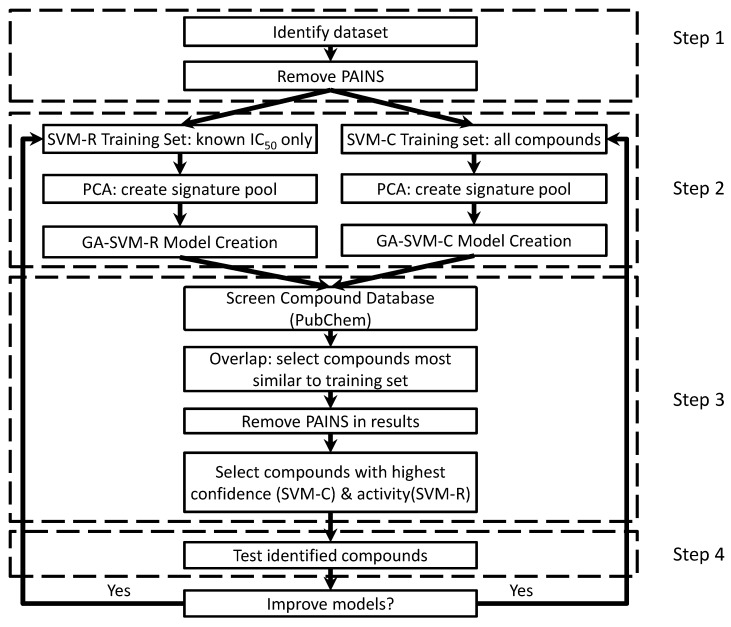
The approach has four main steps: (1) identify a target data set, (2) training predictive classification and QSAR models using the identified data set, (3) screen a compound library with the classification and QSAR models and (4) experimentally validate model predictions. Signature is used to process molecules from the data set or libraries into inputs for our approach. Adapted from Chen, J and Visco, D.P. Identifying novel factor XIIa inhibitors with PCA-GA-SVM developed vHTS models. European J. Med. Chem.; 140:31–41. Copyright ^©^ 2017 Elsevier Masson SAS. All rights reserved.

The approach for this work was previously introduced by the authors [63,64] and is available for reference in Figure 1. One adjustment was made to remove known pan-assay interference compounds (PAINS) [65] and compounds with similar structure from the work using ZINC15 [45]. PAINS are compounds that interact with multiple proteins (i.e., promiscuous) or interact through a variety of different mechanisms (e.g., aggregation, redox, etc.) that are undesirable or atypical of protein/protein interactions. Excluding PAINS and compounds with similar structures remove confounding factors that might affect the performance of any resulting models.

Our approach attempts to leverage existing data using three different ligand-based approaches in concert: (1) active/inactive classification, (2) quantitative structure–activity relationship (QSAR) based activity regression, and (3) similarity to the structure of known ligands. Predictive models are trained using existing experimental and structural data that are then applied in a virtual screening. Scaling linearly with size, the approach can screen small libraries or whole databases. In this work, the entirety of PubChem’s Compound database (currently about 72 million compounds) was screened.

**Figure 2:**
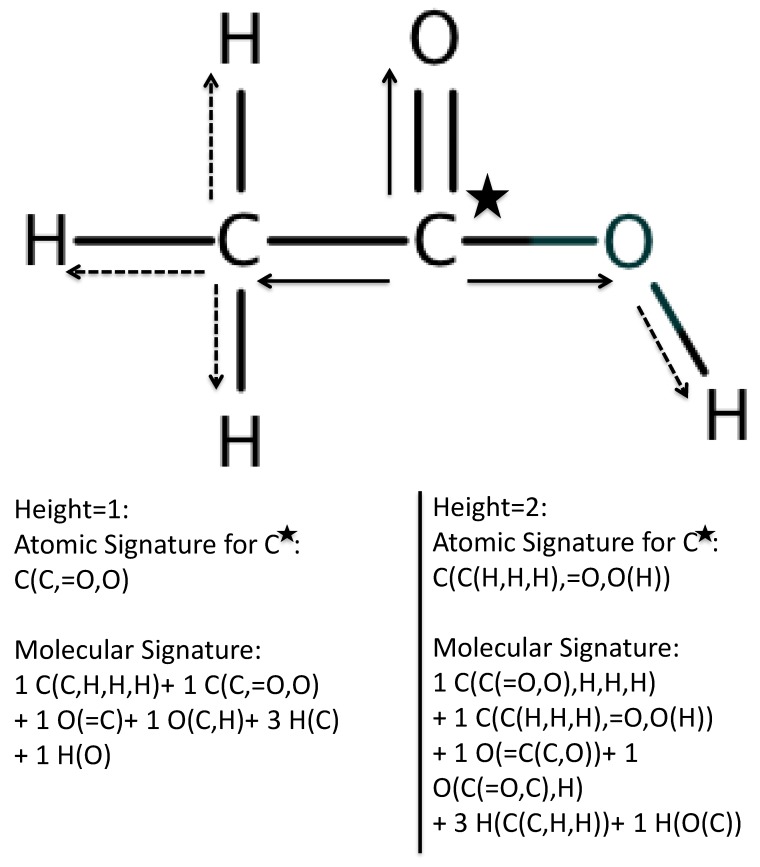
Molecular structure transformation into Signature fragments. Starting from the root atom, like the starred carbon, atomic neighbors and connections, without backtracking, are noted to a pre-determined distance (height) away. When height = 0, only the root atom is noted. When height = 1 (solid arrows), the primary atomic neighbors and their bonds to the root atom are noted. When height = 2 (dashed arrow), the notation from height=1 is amended to include the root atom’s secondary atomic neighbors and their connecting bonds to the primary neighbors. The record for a single root atom is known as an atomic Signature; the collection of atomic Signatures for all atoms in the molecule is known as the molecular Signature. Reproduced from Chen, J and Visco, D.P. Identifying novel factor XIIa inhibitors with PCA-GA-SVM developed vHTS models. *European J. Med. Chem.*; 140:31–41. Copyright ^©^ 2017 Elsevier Masson SAS. All rights reserved.

Three different algorithms were used to create the models for classification and activity prediction: principal component analysis (PCA), support vector machine (SVM) and genetic algorithm (GA). The interaction between the three algorithms is described in *Materials and Methods*. The molecular structure is not directly usable in the algorithms, thus a method of converting molecular structure into numerical data is needed. Signature is a molecular description technique rooted in structure fragmentation [66,67] and elucidation [68]. It was previously used in biological classification and quantitative structure–activity relationship (QSAR) work with the selection of substrates and receptors [63,64,69,70], protein–protein interactions [71] and molecular design of compounds with desired properties [72,73,74,75,76,77]. It is a foundational component of the approach, converting molecules into fragments for our algorithm. Signature molecular fragmentation is demonstrated in Figure 2.

This work is part of a larger effort to determine the effectiveness of the pipeline the authors have previously presented [63,64] when applied to different protein/ligand systems, datasets of various sizes, and data set active/inactive classification distribution. The pipeline has been used to identify inhibitors for Cathepsin L [63] and clotting Factor XIIa [64]. Complement Factor C1s was chosen for this work because of the previously described importance in controlling complement system activation. Additionally, the corresponding dataset, PubChem Bioassay dataset Assay ID (AID) 787 [78], has a relatively small fraction of active compounds (11.8%) and tests the pipeline’s performance when the active:inactive ratio is not close to 1. It should be noted that the pipeline correlates structural feature patterns in compounds with experimental data and applies those correlations, in the form of models, to find new potential ligands. It is not equipped to identify why specific ligands are biologically active while others are not, though speculations can be drawn by correlating atomic Signature to model coefficients as was done in prior work [79].

PubChem Bioassay dataset AID 787 [78] was identified as the data set containing the necessary experimental and ligand structure data for this approach (Figure 1: step 1). PAINS [65] were identified and removed to exclude the addition of confounding variables and data. With a PAINS-free data set, classification and QSAR models were trained and used to screen the entire PubChem Compound database (about 72 million) for potential leads (Figure 1: step 2). Once identified, the potential leads are selected for activity, confidence, and similarity to the training set while removing compounds that may be PAINS (Figure 1: step 3). Experimental verification for biological activity was done using the protocol depositors of AID 787 used [78], scaled up for use to 96 well plates (Figure 1: step 4) to (1) identify new/novel C1s inhibitors, (2) evaluate model predictions and (3) evaluate the pipeline. Based on the results and desired outcomes, models are retrained with the inclusion of new experimental data from the initial validation step to the previous training set. A second round of vHTS and experimental validation was conducted to determine if model performance improved, as was observed in our previous work [63,64].

## 2. Results

### 2.1. First Round Classification and QSAR Model Creation, vHTS and Validation Results

AID 787 contained 183 compounds (23 actives with known IC${}_{50}$ values and 160 inactives) [78]. The maximum candidate concentration tested in the protocol was 50 μM [78]. After PAINS and similarly structured compounds were removed, the data set was reduced to 136 compounds (16 actives and 120 inactives). The PAINS-free training set yielded a total of 1072 atomic Signatures of heights 0, 1, and 2. PCA filtered the atomic Signatures for those contributing significantly towards capturing the observed variance. The filtered atomic Signatures are then used by GA-SVM to build models. Note, two different kinds of models were created using the data available: (1) classification with all PAINS-free data by assigning each compound a class and (2) QSAR-regression with PAINS-free compounds of known IC${}_{50}$ value. Similarity was determined using the “overlap” metric described in *Materials and Methods*. In brief, overlap is the intersection of atomic Signatures in the candidate and the training set in comparison to all atomic Signatures in the candidate. Best model results and statistics are summarized in Table 1.

One item of note is the usage of all atomic Signatures in the SVM-R model. A reason could be the relatively small number of active compounds. With relatively few active compounds, it is possible that all available data is relevant to explaining the variance. Another reason could be the diversity of the atomic Signatures found in the active compounds. If the active compounds share little resemblance, then there might not be a way to differentiate more or less relevant atomic Signatures that capture the observed variance and, thus, all are used.

**Figure 3:**
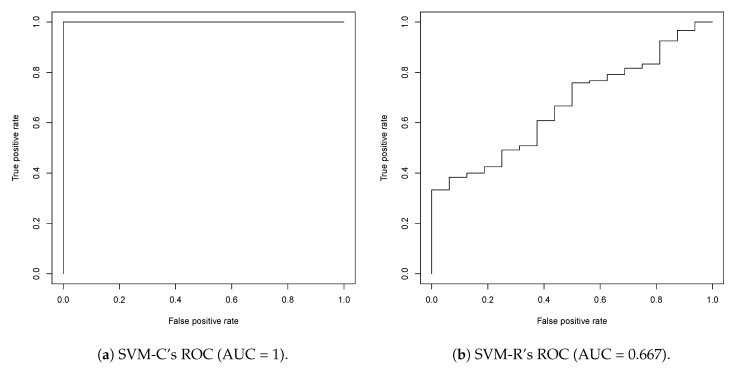
The ROC curves for all 115 SVM-C models and 1 SVM-R model. The curves indicate they both contribute to the identification of active leads: both curves are above the *y* = *x* line, indicating that accurate predictions of active compounds are due to the models and not chance.

Support Vector Machine classification (SVM-C) and regression (SVM-R) model performance was evaluated a priori using receiver operating characteristic (ROC) curves, shown in Figure 3. One hundred fifteen different SVM-C models created but all had the same training error, cross-validation error, and an area under curve (AUC) = 1. Without a non-arbitrary method of identifying a representative model, they were all used in the classification screening. The shape of the ROC curve indicates that there is a sharp division between active and inactive classes during training. It should be mentioned again the SVM-R model performed worse because it was asked to classify compounds with atomic Signatures it was not trained on. Only 16 of the 136 compounds were active and served as the training set for the SVM-R model. When creating the ROC curve, models were used to make predictions for all 136 compounds, which includes the 120 inactive compounds. The 120 inactive compounds contain atomic Signatures that were not present in the SVM-R model training and there is an inverse relationship between prediction accuracy and extrapolation. Therefore, the SVM-R model should perform better than suggested by the ROC curve if extrapolation was limited or eliminated.

Both regression and classification models described in Table 1 were used to screen PubChem’s Compound database (about 72 million). The vHTS results were filtered with the following criteria to create a focused library:

1. Overlap = 1. All atomic Signatures in the candidate molecule are found in the training set.
2. SVM-C score >2 for all 115 SVM-C models. The models must unanimously agree that a molecule should be in the active class and above a certain threshold.
3. Predicted IC${}_{50}$ < 50 μM.

The first criterion is to limit extrapolation and any resulting decrease in predictive power. The second criterion is to prevent arbitrary selection of a representative model because models perform equally well according to the metrics used. Additionally, the threshold value of 2 was used because SVM-C scores indicate a candidates location relative to the active and inactive classes and a larger positive value indicates a greater confidence it will be active [80]. Finally, the third criterion is due to the specification of the protocol used and to focus on the compounds with the highest predicted activity. After applying the criteria, ten compounds were identified but only seven were economically feasible and purchased for experimental validation. Recall that available C1s treatment is a financial burden [20,22,24,25,26], thus economic viability is an important factor for consideration when choosing candidates to test. Four of the seven were ultimately determined to be active for an experimental validation hit-rate of 57%. The seven identified compounds and relevant data are shown in Table 2.

### 2.2. Second Round Classification and QSAR Model Creation, vHTS and Validation Results

The first round hit-rate of 57% is higher than typical HTS hit-rates [38]. Previous work [63,64] has demonstrated an increase hit-rate after retraining models and we follow this approach here as well. Thus, the new experimental data of the seven tested candidates were incorporated into the training set, now containing 143 compounds, and the models were retrained, using the same protocol as in the first round. The results of retraining are detailed in Table 3.

**Figure 4:**
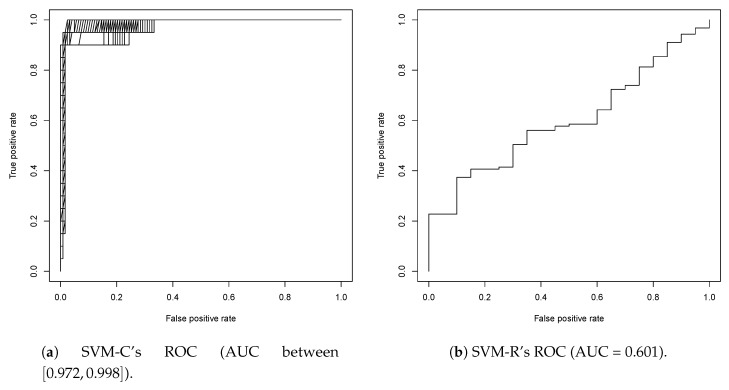
The ROC curves for all 1224 SVM-C models and 1 SVM-R model. Both curves are above the *y* = *x* line, indicating that accurate predictions of active compounds are due to the models and not chance.

SVM-C and SVM-R model performance were again evaluated a priori using ROC curves, shown in Figure 4. Once again, there were multiple SVM-C models created with the same training and cross-validation errors. While AUC can be a way to identify the best model, the AUC values are close enough such that the difference in AUC values is within margins of error. From this, the choice was made to use all 1224 classification models in the screening. As it was previously in the first round, the ROC curve shape indicated a relatively a sharp division between active and inactive classes during training. Additionally, the SVM-R model once again performed worse because it was asked to classify compounds with atomic Signatures it was not trained on, which are the ones found only in inactive compounds in the training set.

Due to the limited number of candidates that passed the previous criteria, it was expected that there would be few compounds that passed the criteria in this round as well. To increase the number of candidates considered, the SVM-C score requirement and the overlap criteria was relaxed. While this does increase extrapolation and the associated error in predictions, more candidates can be identified for further consideration and it serves as an opportunity to evaluate the ability of the pipeline’s models to make accurate predictions when extrapolating. The modified criteria is now:

1. Overlap ≥0.9 i.e., 90% of atomic Signatures in the candidate molecule are found in the training set (modified).
2. SVM-C score >0 for all 1224 SVM-C models. The models must unanimously agree a molecule should be in the active class (modified).
3. Predicted IC${}_{50}<50$ μM.

With the modified criteria, fifty-two compounds were identified for further consideration. After economic considerations, ten of the fifty-two were purchased for validation, five of which were active for a hit-rate of 50%. The results of experimental validation are detailed in Table 4.

## 3. Discussion

### 3.1. Model Discussion

Past work has shown a pattern of an increase in training and cross-validation error between the first and second round, as well as an increase in hit-rates [63,64]. While both errors increased in this current work, the hit-rate dropped slightly. In our previous work [63,64], the expected increase in hit-rate was due to the additional experimental information on candidates containing certain combinations of atomic Signatures, which ultimately focuses the models more towards certain classes of compounds. This effect is likely more pronounced in this work considering the active-inactive compound ratio. The slight drop in hit-rate, then, is likely due to the shift in criteria: to identify more candidates to test, the first two criteria were relaxed between the first and second round to identify more compounds to test. As previously mentioned, there is an inverse relationship between extrapolation and prediction accuracy, and when the second round criteria was relaxed, extrapolation increased. The increase in prediction accuracy, if previous trends are to be believed, combined with the decrease in accuracy due to extrapolation, helps explain the slight drop in hit-rates between the first and second round.

It is interesting that the hit-rates of this study are comparable to our earlier work considering that the fraction of actives in this study was much smaller (0.118) than in previous studies (0.490 [63], 0.563 [64], 0.315 [81]). This is one indication that the pipeline and the models generated are robust and can be applied in situations where there is less data on the desired class, which are the majority of situations in drug discovery.

### 3.2. Complement Factor C1s Inhibitors

In all, seventeen compounds have been identified by the pipeline for further experimental testing. Of the seventeen compounds, nine were determined to be active and can be used in future efforts to study C1s inhibition. As previously stated, the pipeline is not equipped to identify the mechanism these compounds work through, but structure comparisons of the results do yield some speculations as to the roles of different functional groups.

**Figure 5:**
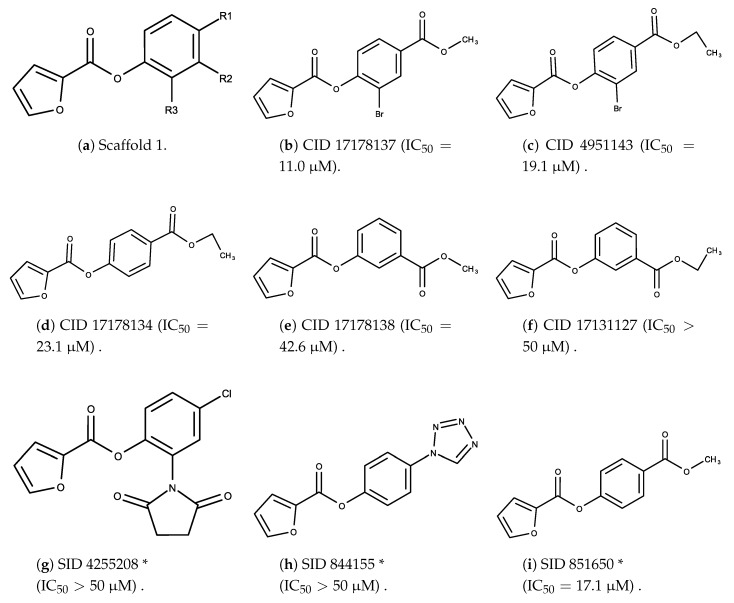
Scaffold 1 and the nine compounds containing the scaffold. This scaffold was only found in three compounds in the original data set, denoted with an asterisk (*). CID = PubChem Compound ID, SID = PubChem Substance ID.

**Figure 6:**
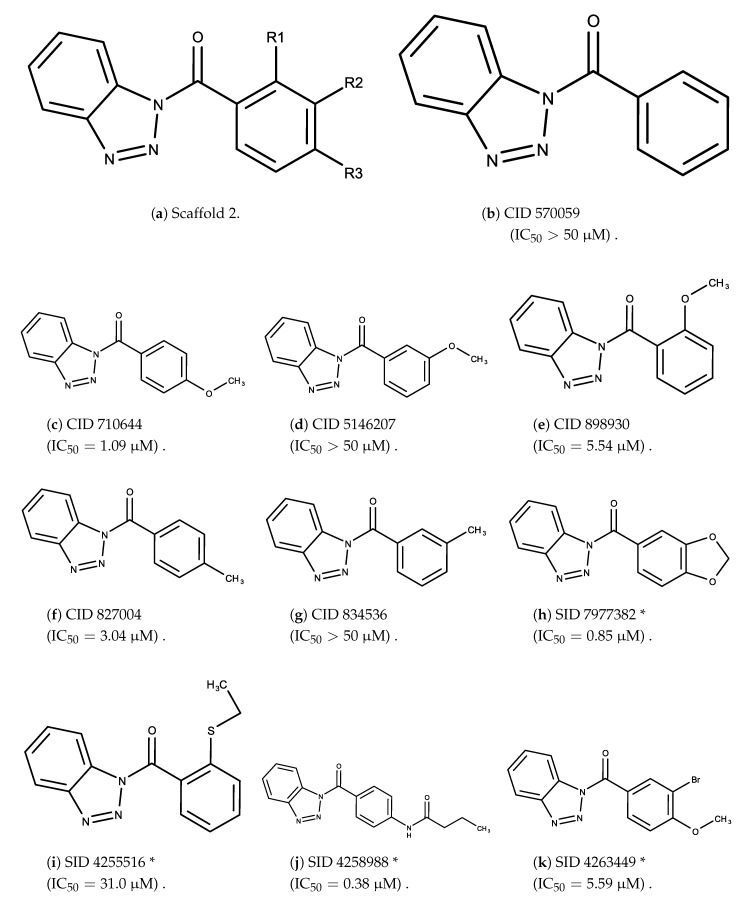
Scaffold 2 and ten of the thirteen compounds containing the scaffold. This scaffold was only found in four compounds in the original data set, denoted with an asterisk (*). CID = PubChem Compound ID, SID = PubChem Substance ID.

The candidates can largely be split into two different groups: those with the scaffold shown in Figure 5a (five compounds in all) and those with the scaffold shown in Figure 6a (nine compounds in all). The compounds were a mixture of active and inactive compounds that allows for more meaningful comparisons on the impact of different functional groups.

When examining the compounds containing scaffold 1 in Figure 5, several trends emerge:

1. The bromine group in the ortho position seems to have a smaller impact on activity. Structures shown in Figure 5c,d are identical except for the bromine group and the IC${}_{50}$ values are similar as well (19.1 μM vs. 23.1 μM, respectively). A similar analysis can be made for the structures shown in Figure 5b,i (11.0 μM vs. 17.1 μM, respectively).
2. The position of the larger functional groups has a larger impact on activity. Structures in Figure 5d,f are the same except the ester group is shifted from the para to the meta position. However, this is the difference between active and inactive. A similar analysis can also be made using the structures in Figure 5b,e. If the Br group is assumed to have a small or minimal effect (as mentioned above), then the difference in activity can be accounted by the shift of the ester group from the para to the meta position. Thus, perhaps the para position is preferable to the meta position.
3. The identity of the functional group is important. PubChem Substance ID (SID) 4255208 and 844155, shown in Figure 5g,h, have very different functional groups than the rest of the structures shown in Figure 5. The previous conjecture for functional group placement at the ortho and para positions suggests that the inactivity is due to the identity of the functional groups rather than the position of them.

Examining six of the nine compounds containing scaffold 2 that selected for testing and four compounds containing scaffold 2 in the training set also yielded interesting patterns:

1. The bromine group in the meta position seems to have a smaller impact on activity. Structures shown in Figure 6c,k are identical except for the bromine group and the IC${}_{50}$ values are similar as well (1.09 μM vs. 5.59 μM, respectively).
2. The position of the large functional group greatly affects activity. Structures in Figure 6c–e are the same structure with the ether group at para, meta, and ortho positions. The ether group at the para position is most active, then ortho and, finally, meta. A similar respective pattern can also be seen with structures in Figure 6f,g and mirrors the pattern seen with scaffold 1. As a reference, Figure 6b, which is scaffold 2 with H at all substitution positions, is inactive so any activity and variance in activity are due primarily to the presence and position of the functional group and not the scaffold.
3. The identity of the functional group is important. CID 898930 and SID 4255516, shown in Figure 6e,i have functional groups that occupy the the same ortho position. However, there is a big difference in activity (5.54 μM vs. 31.0 μM, respectively). The difference in activity is likely due to the identity of the functional group. A similar analysis can be done of CID 710644 and SID 4258988, shown in Figure 6c,j. While the difference in activity is not a big (1.09 μM vs. 0.38 μM, respectively), the observed difference is likely due to the identity of the functional group at the para position.
4. There may be a compensatory effect of having functional groups at the meta and para positions. When methoxy groups at the meta and para positions fuse into a dioxol ring as seen in SID 7977382 from the training set (Figure 6h), the resulting compound is also active. This suggests a compensatory effect of having a ring or having groups at both the meta **and** para positions instead of just methoxy groups at the para **or** meta positions (as in Figure 6c,d, respectively.)

## 4. Materials and Methods

### 4.1. Creating/Training Predictive Models

After identifying data sets containing relevant information on receptor–compound interactions, the structures of the tested compounds are converted into atomic Signatures such as the ones detailed in Figure 2. The atomic Signatures are then used to create and train models using a previously presented integrated PCA, GA, and SVM approach [63,64]. To summarize, the weighted contributions of each atomic Signature in principle components created by PCA is used to identify the atomic Signatures that contribute the most to capturing variance. In this way, the physical significance of atomic Signatures is retained while eliminating atomic Signatures, and thus extra variables, for the proceeding steps. The identified atomic Signatures are then used to create GA-SVM vHTS models.

When creating the vHTS models, the interaction between GA [82] and SVM [83] enables the robust testing of different atomic Signature combinations to identify an optimum subset to use. GA [82] creates many different atomic Signature combinations that are then used to create SVM models. The SVM models are evaluated for cross-validation accuracy and reported back to GA as the score for the atomic Signature combinations. GA then implements genetic operations to select the best atomic Signature combinations, create new combinations from old ones, and to perturb the best combinations to test for robustness.

SVM [83] models data in as many dimensions as there are variables so the removal of atomic Signatures that capture minimal amounts of variance reduces the complexity of the SVM models created. Depending on the kind of data available, SVM will create classification models with discrete data (e.g., +/−) and regression with continuous data (e.g., IC${}_{50}$). Cross-validation was used as the scoring metric mainly as a method to measure predictive power. Cross-validation is a technique where data is segregated into training and test sets for model development and evaluation, respectively. When evaluating data it was not trained with, model accuracy can be interpreted as a measure of its predictive power and, because it is desired in vHTS, a good scoring metric in model development.

In modeling, always be wary of overfitting, or the capturing and description of the variance and the noise in data, in modeling. To minimize and mitigate chances of overfitting, several different features of the approach are used. Firstly, the reduction in the atomic Signatures considered in GA-SVM model creation for simplifying the resulting models also removed atomic Signatures that would be used for overfitting. Secondly, the nature of GA allows for the vigorous testing of many different models, conditions, and atomic Signature combinations to yield the most optimum and robust model. Finally, the cross-validation approach sequesters a portion of the data to be used only for model evaluation and overfitted models should perform poorly when applied. Overfitting is a constant concern, which is why safeguards were developed and implemented.

### 4.2. Screening with Predictive Models

Once trained, the vHTS models are implemented on compound databases, like PubChem Compound in this work, to identify potential candidates for experimental validation. Models are evaluated a priori to identify likely successful vHTS models and a posteriori to determine how successful the approach was in identifying candidates. Accuracy is used a priori to identify the vHTS models most likely to succeed in identifying candidates and is defined by Weis [80] as:

$$Accuracy=\frac{TP+TN}{TP+TN+FP+FN}$$

where “TP” and “TN” mean true positive and true negative, respectively, and “FP” and “FN” mean false positive and false negative, respectively. Cross-validation accuracy was the primary metric used as a measure of predictive power, which is most desired in vHTS. Training accuracy is used as a secondary metric to decide between models that have the same cross-validation accuracy. Precision is an a posteriori metric of vHTS success in identifying candidates and is defined by Weis [80] as:

$$Precision=\frac{TP}{TP+FP}$$

where “TP” and “FP” mean true positive and false positive. Hit-rate, a commonly used term in HTS, has the same definition.

Prediction power is inversely related to extrapolation: predictions are more accurate with less extrapolation and vice versa. This relationship has also been verified in previous Signature vHTS work [80]. While similarity can be measured in different ways [84], the “overlap” metric, based on the set-theoretic definition of the Tanimoto Coefficient [84] and defined by Weis [80], is used in this work:

$$\Omega =\frac{x_{\left[min,max\right]}}{x_{total}},$$

where $x_{\left[min,max\right]}$ is the total number of unique atomic Signatures in the compound that falls within the maximum and minimum occurrences observed in the training set.

### 4.3. Model Generation Parameters

All modeling processes were done on dual Intel Xeon processors (E5-2697W, 3.10 GHz, 48 independent threads). The 48 independent threads were utilized to run 48 iterations of model generations with different initial conditions. Additionally, 32 of the 48 independent threads were used to split the screening load by splitting the PubChem Compound database (72 million compounds) into 32 subsets and each subset screened on its own thread. PCA, GA and SVM were done using R Statistical Software: PCA using the “eigen” function, GA using the “ga” function in the “GA” package [85], and SVM using the “ksvm” function in the “kernlab” package [86]. Parameters for GA were as follows: elitism rate = 0.7, crossover rate = 0.8, mutation rate = 0.1, population size = 1000, maximum iterations = 1000, stop after 100 iterations of no improvement. Parameters for SVM were as follows: cost ranges from 0.01 to 1 with step size 0.01, 10 fold cross-validation, ν = 0.2, linear kernel.

### 4.4. Fluorescent Complement Factor C1s Inhibitor Screening Assay

#### 4.4.1. Assay Materials

Assay buffer: H${}_{2}$O with 50 mM HEPES, 200 mM NaCl, 0.2% polyethylene glycol (PEG) and adjusted to pH 7.5. Enzyme Solution: activated human complement factor C1s (final concentration 0.02 mg/mL). Substrate Solution: Boc-Leu-Gly-Arg-AMC (final concentration 15 μM). Plate: Corning black polystyrene 96-well, flat bottom. HEPES (H4034), PEG (P3390), and Corning flat-bottom, black polystyrene 96 well plates (CLS3915) are purchased from Sigma Aldrich (St. Louis, MO, USA), NaCl from Chem-Impex (CatID 00829; Wood Dale, IL, USA) C1s from CalbioChem (CatID 204879; Billerica, MA, USA), and the Boc-Glu-Ala-Arg-AMC from Bachem (CatID I-1105; Torrance, CA, USA). All testing compounds were purchased via Molport (Riga, Latvia).

#### 4.4.2. Assay Protocol

1. Serial dilute identified compounds at 50× concentration in DMSO: eight four-fold dilutions from 2.5 mM to 152.6 nM. Final testing concentration from 50 μM to 3.05 nM.
2. Fill 96 well plate with 50 μL substrate solution except column 10.
3. Add 2 μL of the compound from step 1 to corresponding wells in the plate.
4. Add 50 μL enzyme to all wells with compound added and column 10.
5. Control: Add 50 μL enzyme to column 12.
6. Blank: Add 50 μL buffer to column 11.
7. Protein Check: Add 50 μL buffer to column 10.
8. Incubate for 2.5 h at room temperature.
9. Read fluorescence (excitation 355, emission 460) on Tecan M200.

#### 4.4.3. Percent Inhibition Determination

Percent inhibition was calculated using the following relationship:

$$\%inhibition=(1-\frac{signal-\bar{blank}}{control-\bar{blank}}*100).$$

To calculate the IC${}_{50}$ value, a linear interpolation of the closest data point above and below 50% inhibition was conducted. If 50% inhibition was not included in the range of inhibition values, the compound was deemed inactive. There were no compounds for which the IC${}_{50}$ value was below the minimum testing concentration.

## 5. Conclusions

The complement system is one of the key parts of the innate portion of the immune system. It targets material for removal, recruits macrophages, upregulates pro-inflammatory signals, and activates the pathways to create membrane attack complexes. Dysregulation of any step in the system can result in undesired activity and is implicated in many different diseases and disorders. Complement factor 1, as one of the main ways to initiate the system, is a good target for treatment. Complement factor 1 is composed of three different parts with different roles. C1q is responsible for the target recognition and activates C1r. Next, C1r cleaves and activates C1s, which then activates the following steps in the complement system. C1q’s recognition ability is key for the immune system and its inhibition is not desirable as a treatment target. C1r is an intermediate step while C1s is the activator of subsequent steps. Thus, C1s is more desirable than C1r as a target and was the focus of the work presented.

There are currently two different kinds of marketed treatment: donated C1 inhibitor derived from an external source or antibodies. Previous attempts have identified small molecule inhibitors but have not been marketed. To identify new small molecule inhibitors that can be marketed, computational techniques previously presented and used to identify other inhibitors were applied here to find seventeen small molecule inhibitor candidates in two rounds: seven in the first round and ten in the second round. Four of the seven compounds in the first round were active for a hit-rate of 57% and five of the ten compounds in the second round were active for a hit-rate of 50%. The hit-rate dropped slightly between the first and second round, but this was likely due to the extrapolation necessary to identify enough compounds for testing in the second round.

The molecular structures of the candidates and the compounds composing AID 787 were analyzed to identify different avenues for additional study. Two different scaffolds were identified and structure–activity relationships of some noteworthy functional groups were determined. The scaffolds could be optimized for better metabolism and pharmacokinetic properties using known and newly learned structure–activity relationship information. Additionally, the structure–activity relationship information learned could be used to identify potential new scaffolds to examine and/or interrogate the functionality of the binding pocket.

Finally, the work presented here is one part of a series to investigate the applicability of the pipeline for use in vHTS across a variety of dimensions such as system size, active/inactive classification distribution and value of model retraining. Collectively, the results of this work and others will guide future applications of the pipeline to help drug discovery efforts and resource utilization.

## Figures and Tables

**Table 1:** First round model results and statistics.

	**SVM-C**	**SVM-R**
**Training set** **NO PAINS**	136 compounds (16 active, 120 inactive)	16 compounds (all active)
**Training set** **atomic Signatures**	11 h = 0; 136 h = 1; 925 h = 2; 1072 total	8 h = 0; 57 h = 1; 165 h = 2; 230 total
**Principal Component** **Analysis results**	159 of 1072 atomic Signatures	230 of 230 atomic Signatures
**Models Created**	115	1
**Training Error**	0	0
**Cross-Validation** **Error**	0.007	0.090

**Table 2:** vHTS first-round validation results. Compounds selected are commercially available, economically viable, and passed the following criteria: predicted $IC_{50}<50$ μM, SVM-C scores >2 for all 115 SVM-C models, and overlap = 1. Compounds were tested in triplicate. Compounds  with reported IC${}_{50}$ values are active across the triplicates. The reported $IC_{50}$ value is the mean of the triplicates. CID is the compound’s PubChem ID number. * Compound showed weak activity at 50 μM but is inactive under stated criteria.

Structure	CID	Predicted IC${}_{50}$[μM]	Experimental IC${}_{50}$[μM]
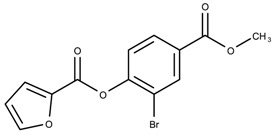	17178137	21.9	11.0±1.08
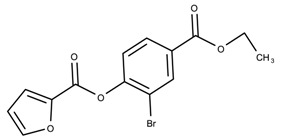	4951143	6.77	19.1±2.73
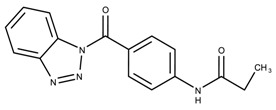	2986934	1.18	0.34±0.14
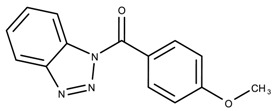	710644	4.36	1.09±0.55
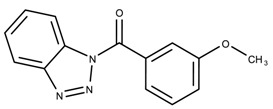	5146207	10.9	>50 *
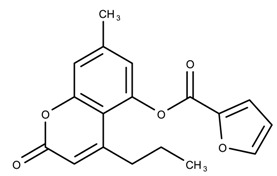	807111	8.88	>50 *
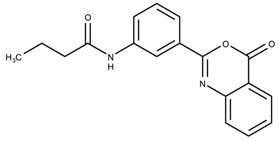	1107361	11.2	>50

**Table 3:** Second round model results and statistics.

	**SVM-C**	**SVM-R**
**Training set** **NO PAINS**	143 compounds (20 active, 123 inactive)	20 compounds (all active)
**Training set** **Signatures**	11 h = 0; 136 h = 1; 925 h = 2; 1072 total	8 h = 0; 57 h = 1; 165 h = 2; 230 total
**Principal Component** **Analysis results**	164 of 1072 atomic Signatures	186 of 230 atomic Signatures
**Models Created**	1224	1
**Training Error**	0.021	0.108
**Cross-Validation** **Error**	0.020	0.162

**Table 4:** vHTS second round validation results. The compounds selected are commercially available, economically viable and passed the following criteria: predicted IC${}_{50}<50$ μM, SVM-C scores >0 and overlap >0.9. Compounds are tested in triplicate. Compounds with reported $IC_{50}$ values are active across the triplicates. The reported IC${}_{50}$ value is the mean of the triplicates. CID is the compound’s PubChem ID number, * Compound showed weak activity at 50 μM but is inactive under stated criteria.

Structure	CID	Predicted IC${}_{50}$[μM]	Experimental IC${}_{50}$[μM]
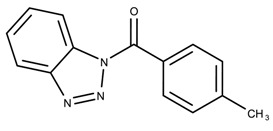	827004	0.30	3.04±1.24
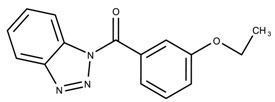	4957387	4.27	32.9±3.04
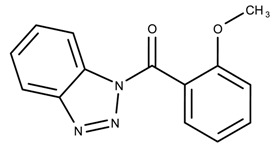	898930	26.01	5.54±1.19
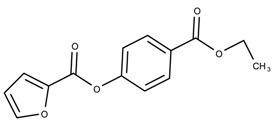	17178134	7.21	23.1±1.39
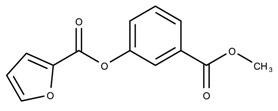	17178138	33.44	42.6±0.72
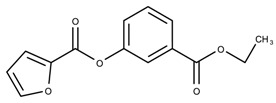	17131127	23.71	>50 *
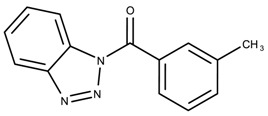	834536	1.66	>50 *
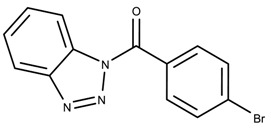	693001	0.43	>50 *
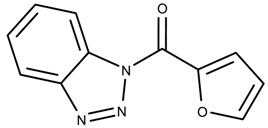	792914	2.05	>50 *
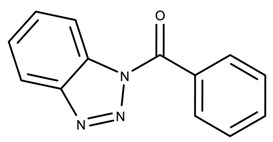	570059	4.20	>50 *

## Supplementary Materials

Supplementary File 1
