# Supplementary material for: Pharmaceutical Machine Learning: Virtual High-Throughput Screens Identifying Promising and Economical Small Molecule Inhibitors of Complement Factor C1s
Source: Biomolecules. 2018 May 7;8(2):24. doi: 10.3390/biom8020024 (PMC6023033; doi:10.3390/biom8020024)
Supplement: Supplementary File 1 [file biomolecules-08-00024-s001.pdf]

Supplementary Materials: Pharmaceutical Machine Learning: Virtual High-Throughput Screens Identifying Promising and Economical Small Molecule Inhibitors of Complement Factor C1s.

Jonathan Jun Feng Chen [1,†], Lyndsey N. Schmucker [2,†] and Donald P. Visco Jr [3,†,*]

Table S1. Training Data. All data used to train the first-round classification and regression vHTS model. The same data is augmented with the *in vitro* experimental results and used to train the second-round classification and regression vHTS models. SID= PubChem Substance ID

**Structure**	SID	Classification	Activity $IC_{50}[\mu M]$
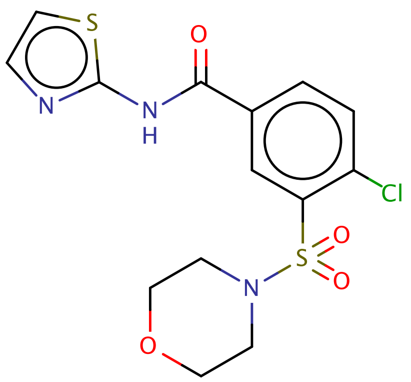	3711963	Inactive	N/A
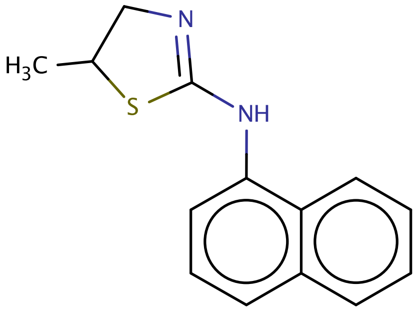	3712600	Inactive	N/A

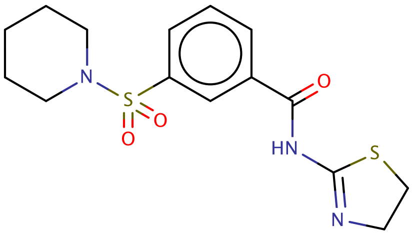	3712681	Inactive	N/A
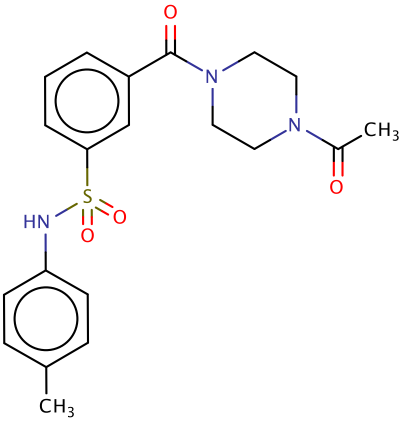	3712850	Inactive	N/A
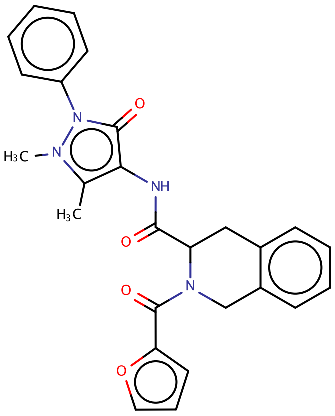	3713318	Inactive	N/A

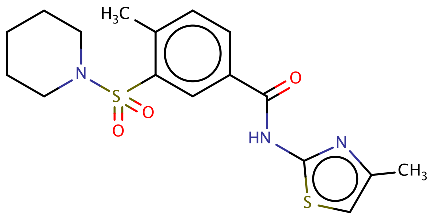	3713502	Inactive	N/A
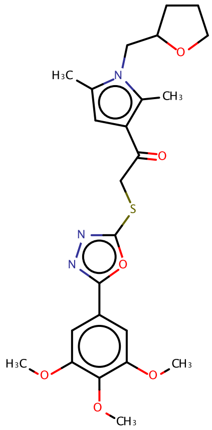	3714494	Inactive	N/A
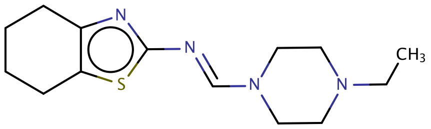	3715220	Inactive	N/A

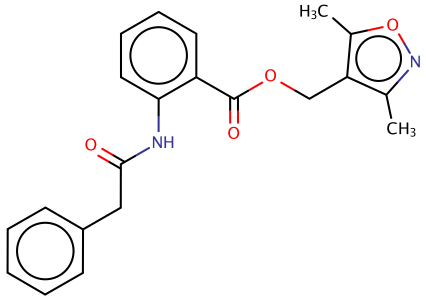	3715428	Inactive	N/A
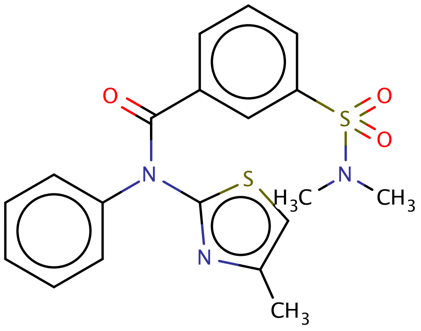	3715753	Inactive	N/A
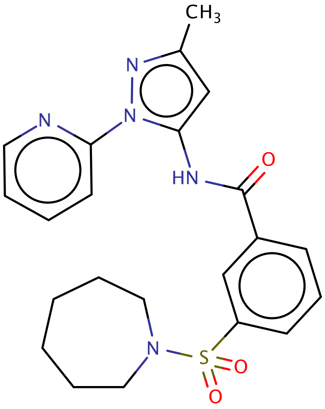	3715870	Inactive	N/A

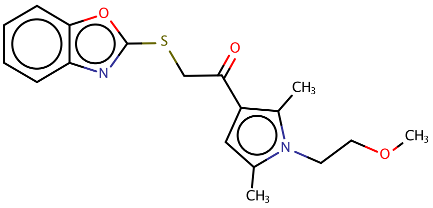	3716178	Inactive	N/A
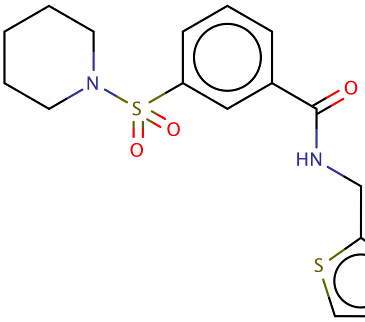	3716847	Inactive	N/A
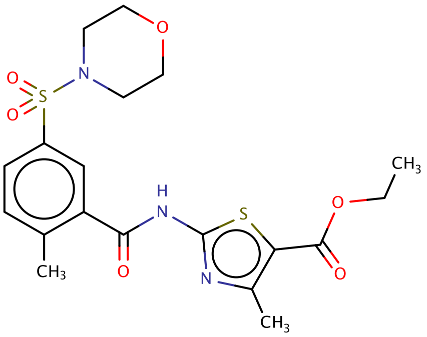	3717180	Inactive	N/A

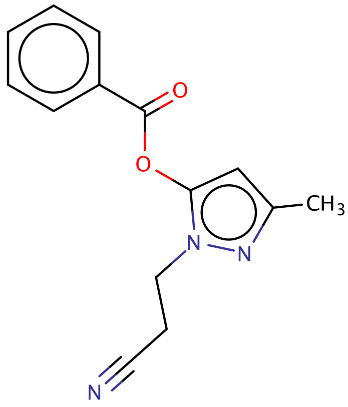	3717586	Active	2.14
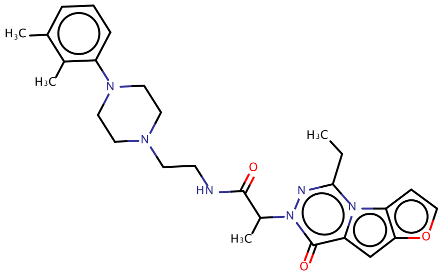	4240573	Inactive	N/A
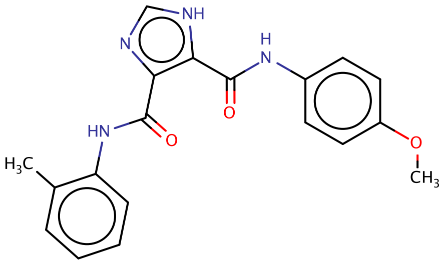	4241692	Inactive	N/A

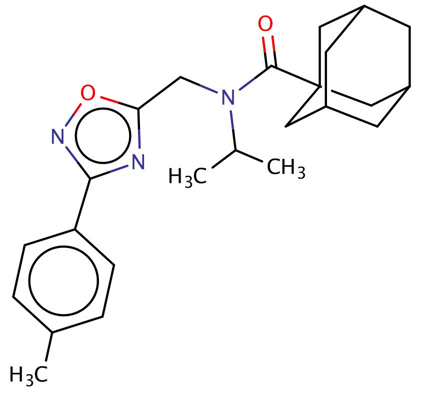	4242011	Inactive	N/A
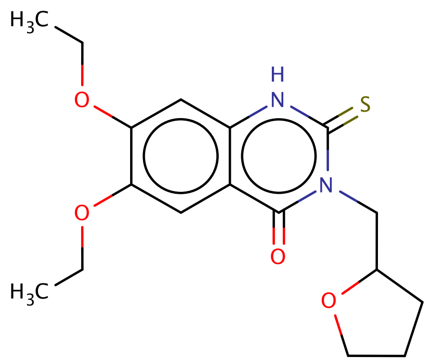	4242319	Inactive	N/A
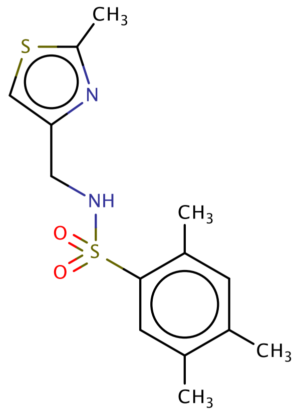	4243864	Inactive	N/A

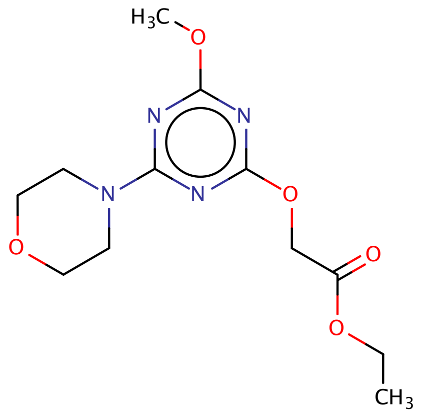	4244211	Inactive	N/A
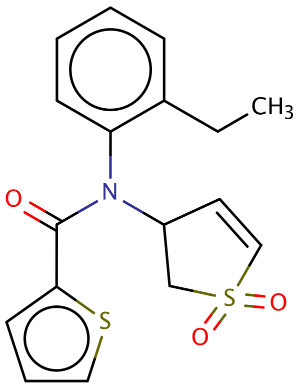	4245215	Inactive	N/A
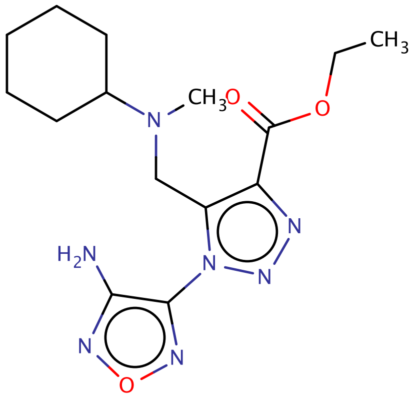	4246549	Inactive	N/A

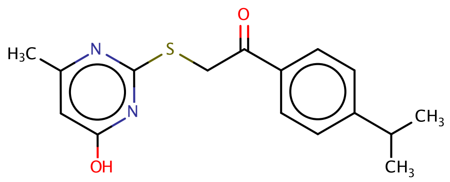	4246823	Inactive	N/A
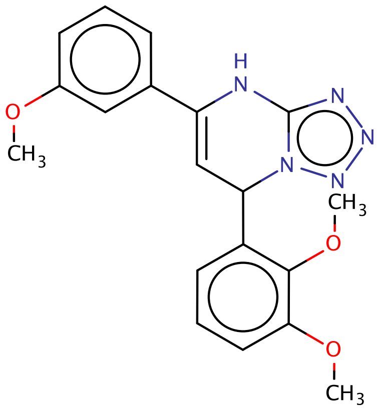	4246912	Inactive	N/A
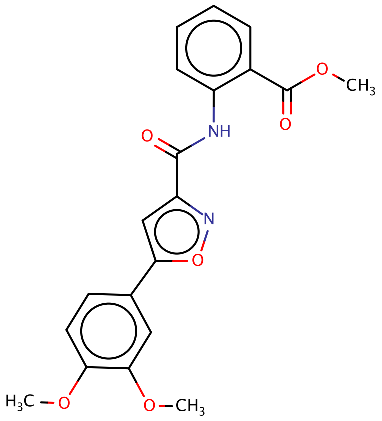	4247384	Inactive	N/A

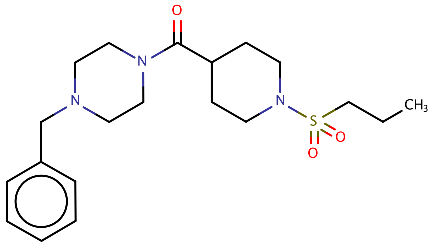	4248033	Inactive	N/A
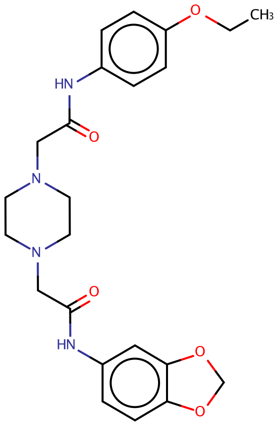	4248495	Inactive	N/A
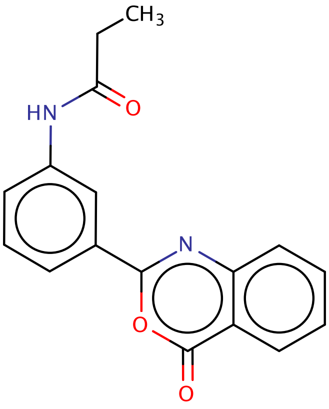	4249007	Active	34.72

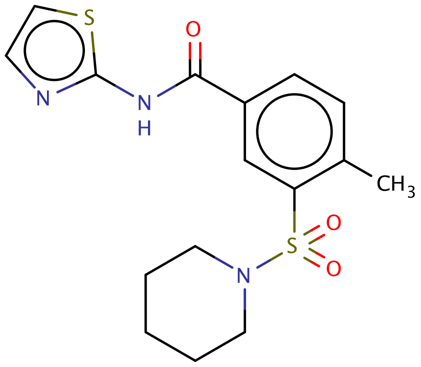	4249302	Inactive	N/A
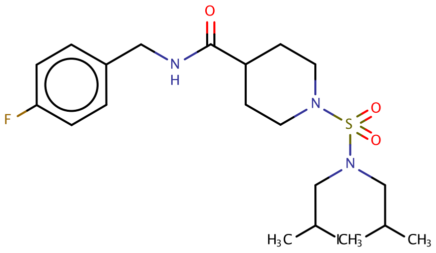	4250394	Inactive	N/A
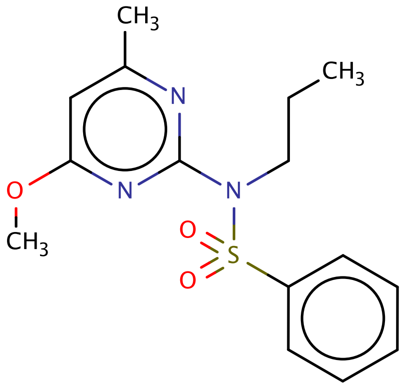	4251421	Inactive	N/A

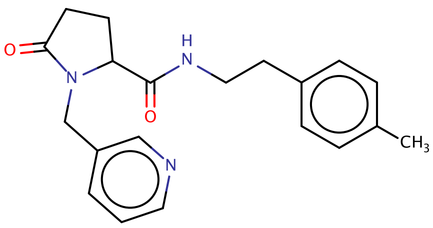	4251548	Inactive	N/A
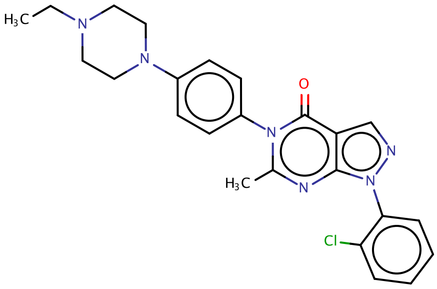	4251738	Inactive	N/A
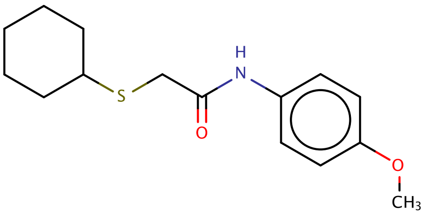	4254582	Inactive	N/A

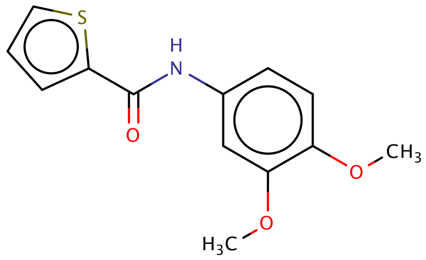	4254762	Inactive	N/A
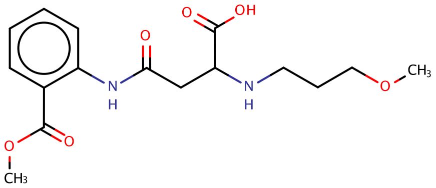	4254843	Inactive	N/A
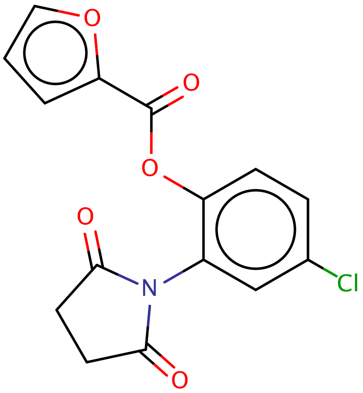	4255208	Inactive	N/A

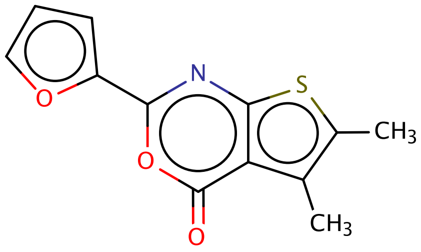	4255209	Inactive	N/A
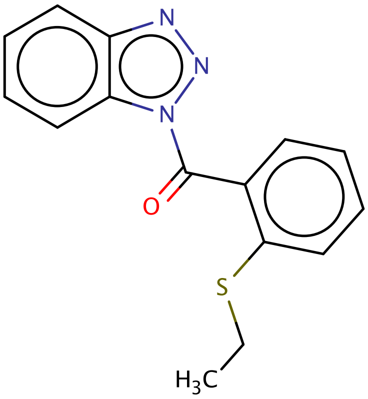	4255516	Active	30.99
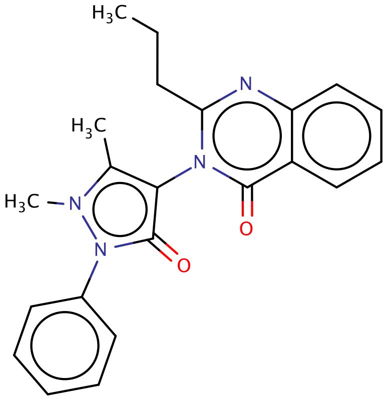	4255537	Inactive	N/A

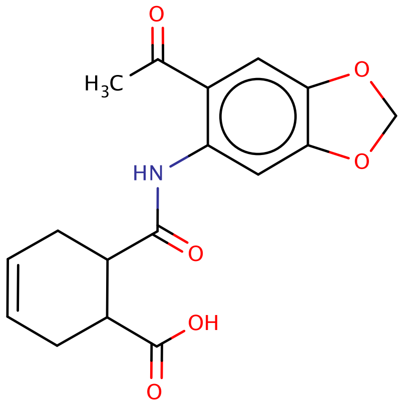	4255573	Inactive	N/A
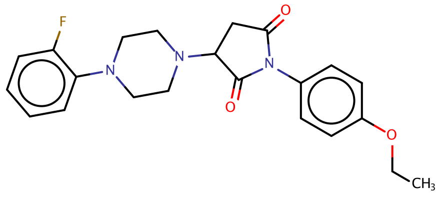	4255800	Inactive	N/A
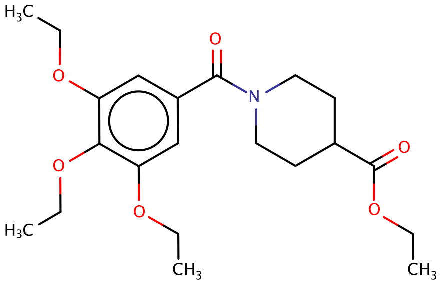	4256088	Inactive	N/A

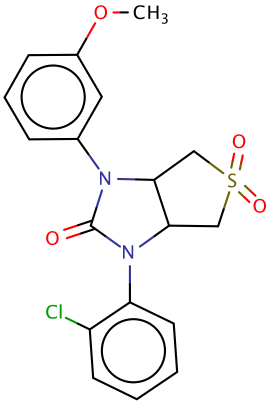	4256347	Inactive	N/A
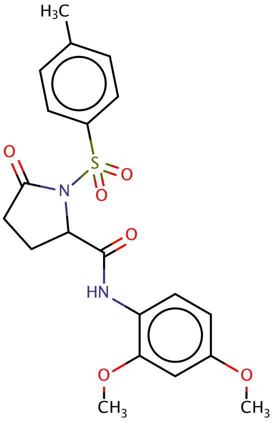	4256651	Inactive	N/A
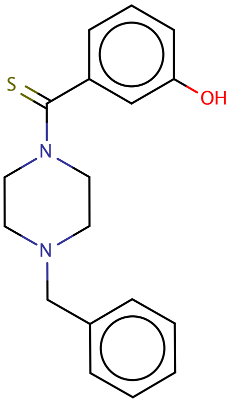	4256969	Inactive	N/A

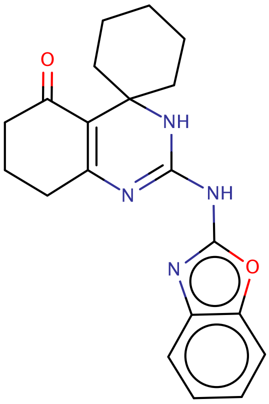	4257420	Inactive	N/A
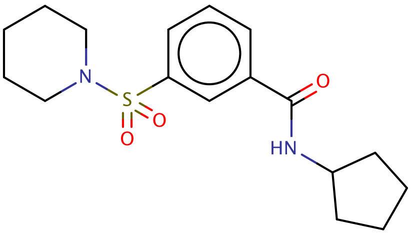	4257713	Inactive	N/A
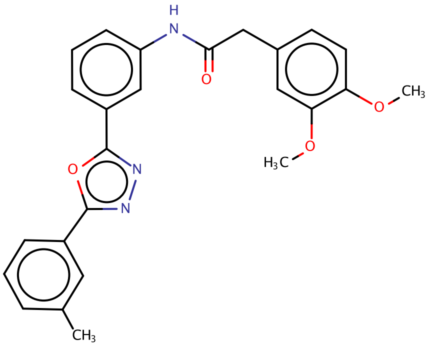	4257844	Inactive	N/A

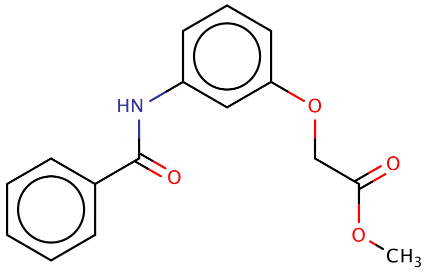	4258135	Inactive	N/A
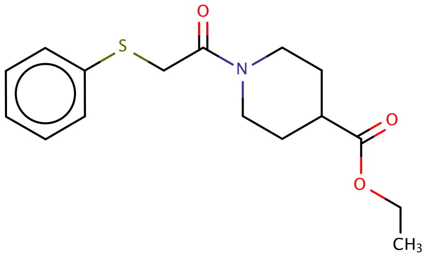	4258729	Inactive	N/A
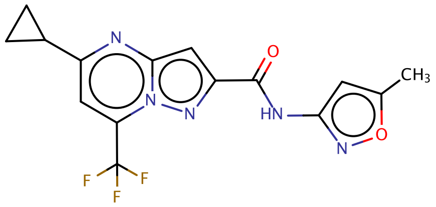	4258821	Inactive	N/A

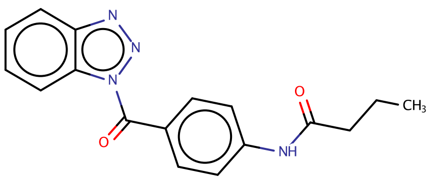	4258988	Active	0.38
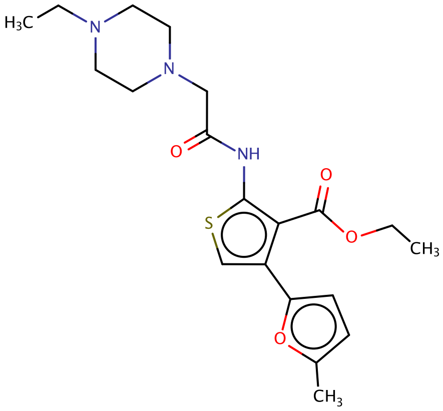	4259517	Inactive	N/A
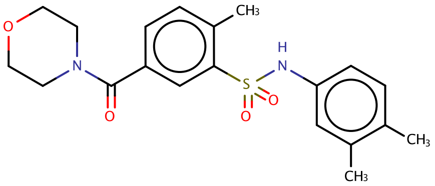	4259584	Inactive	N/A

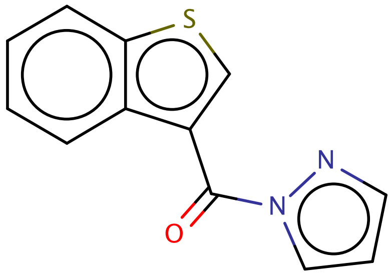	4259744	Active	14.91
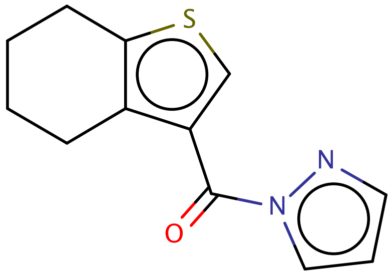	4260000	Active	3.83
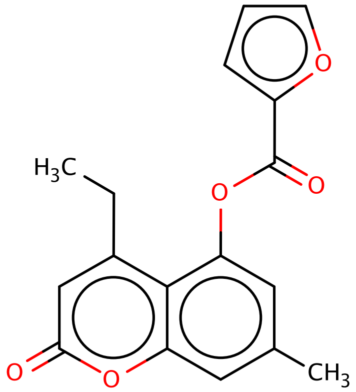	4260130	Active	10.1

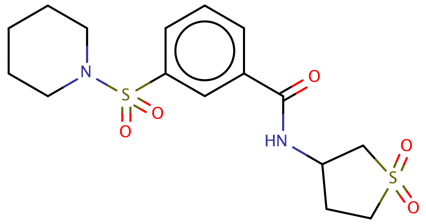	4261230	Inactive	N/A
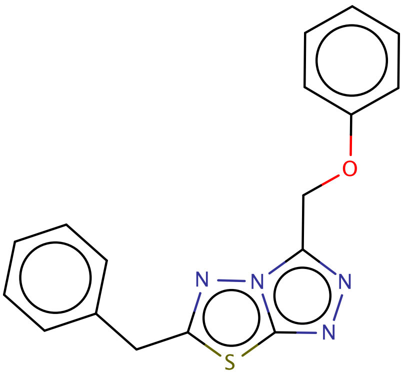	4261270	Inactive	N/A
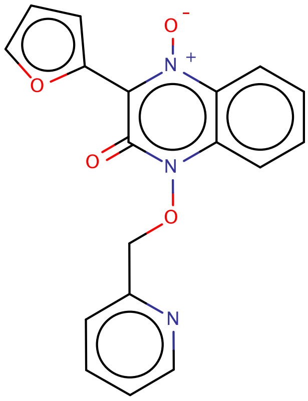	4261321	Active	37.87

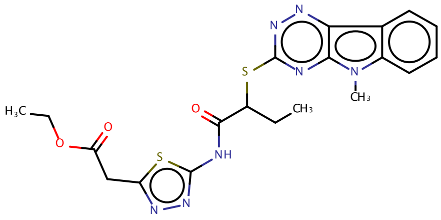	4261362	Inactive	N/A
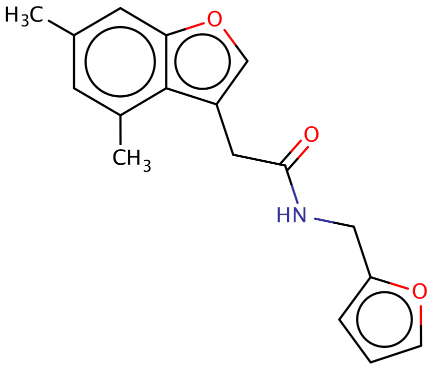	4261432	Inactive	N/A
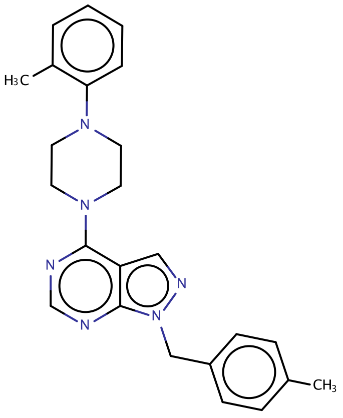	4261564	Inactive	N/A

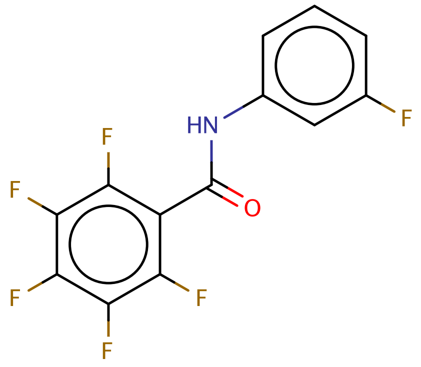	4261661	Inactive	N/A
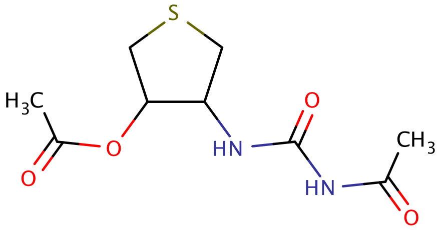	4261818	Inactive	N/A
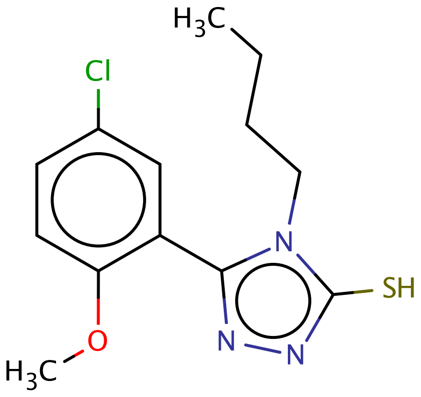	4261899	Inactive	N/A

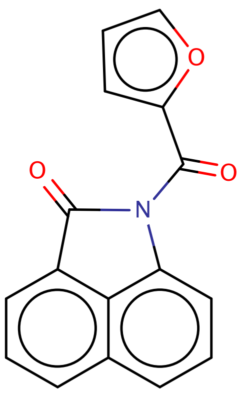	4262775	Inactive	N/A
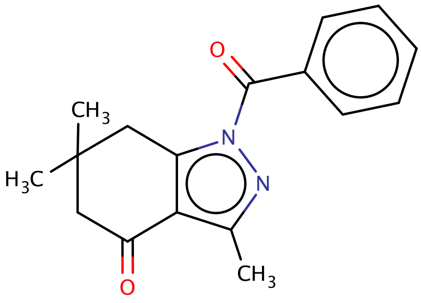	4263151	Active	5.35
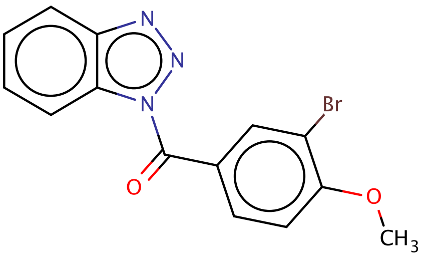	4263449	Active	5.59

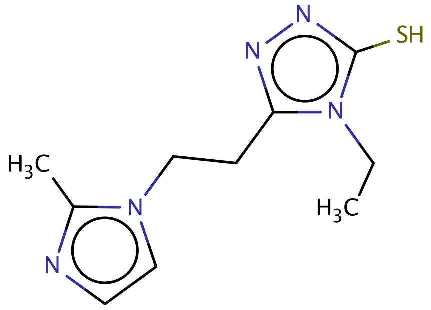	4263981	Inactive	N/A
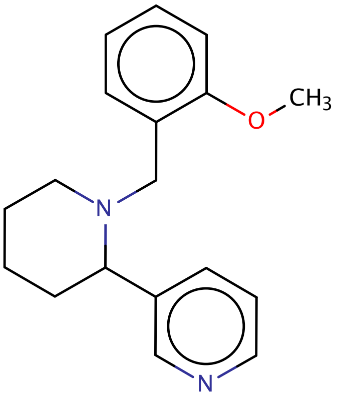	4264329	Inactive	N/A
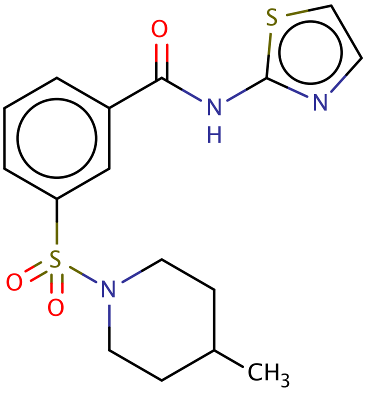	4264469	Inactive	N/A

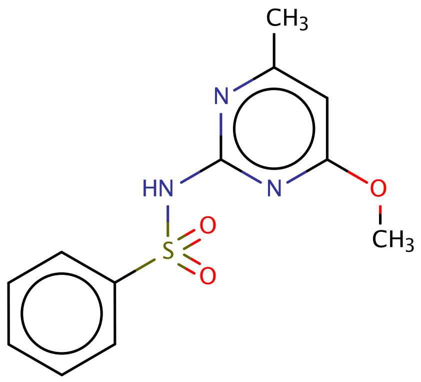	4264491	Inactive	N/A
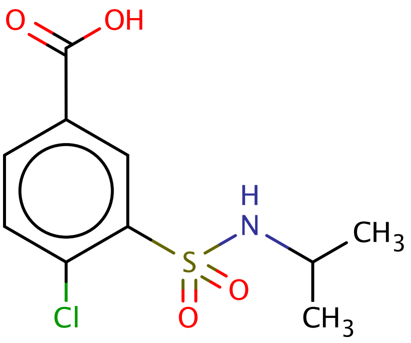	4264513	Inactive	N/A
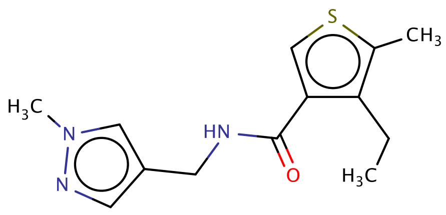	4265032	Inactive	N/A

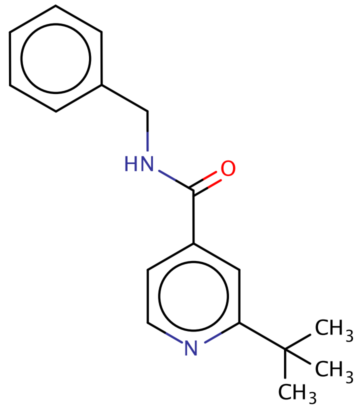	4265342	Inactive	N/A
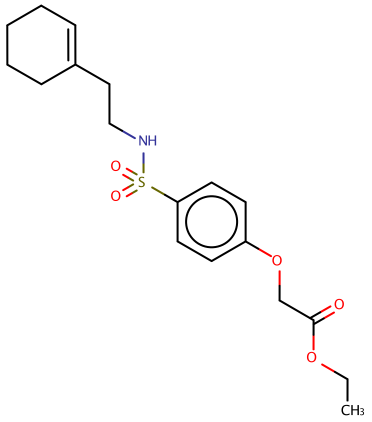	4265617	Inactive	N/A
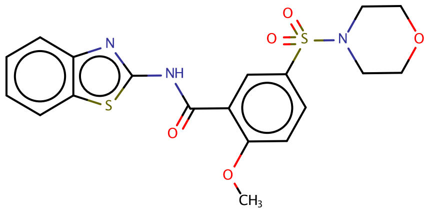	4265630	Inactive	N/A

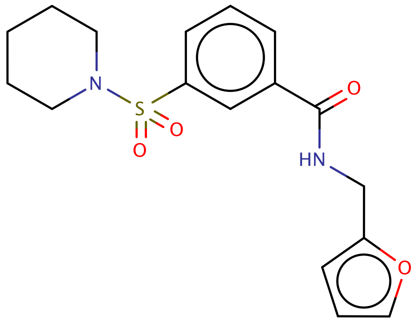	4265764	Inactive	N/A
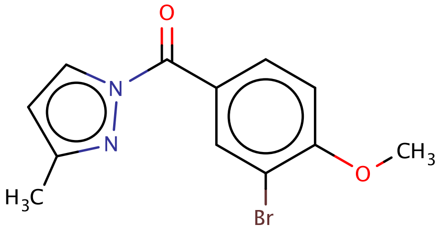	4265870	Inactive	N/A
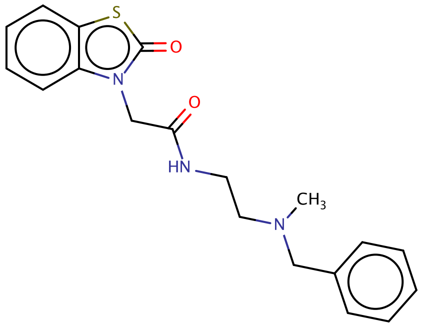	7965115	Inactive	N/A

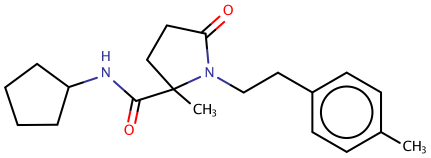	7965493	Inactive	N/A
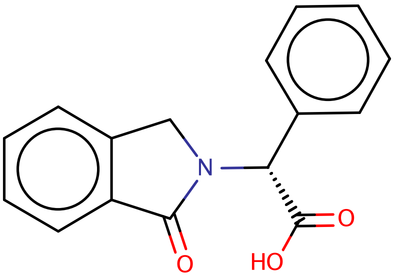	7966122	Inactive	N/A
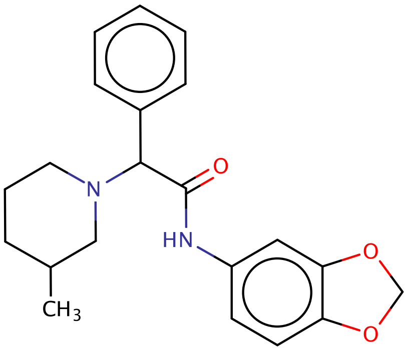	7967020	Inactive	N/A

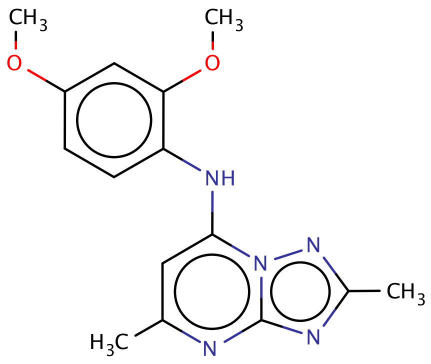	7969138	Inactive	N/A
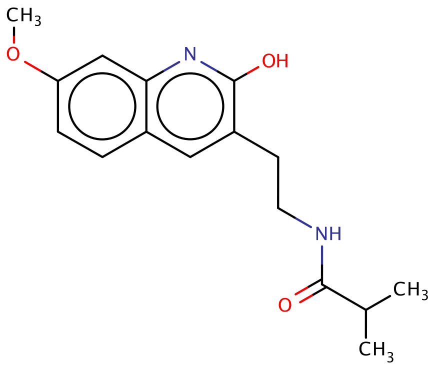	7971047	Inactive	N/A
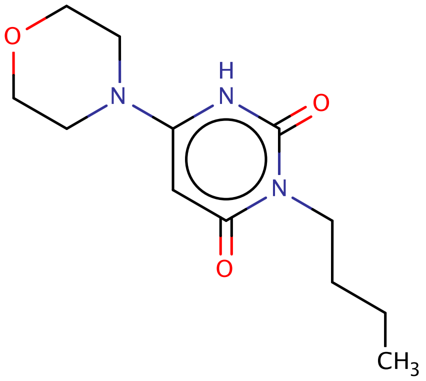	7971114	Inactive	N/A

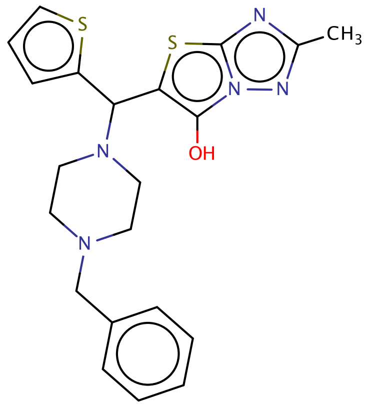	7971311	Inactive	N/A
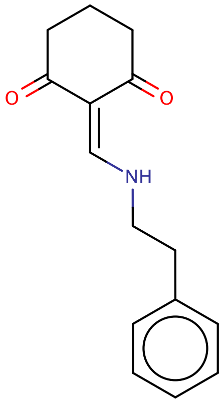	7972455	Inactive	N/A
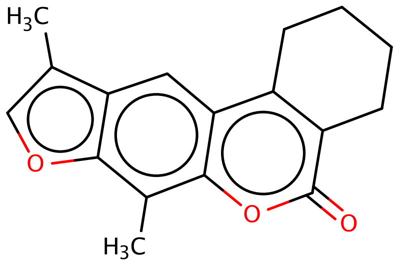	7972585	Inactive	N/A

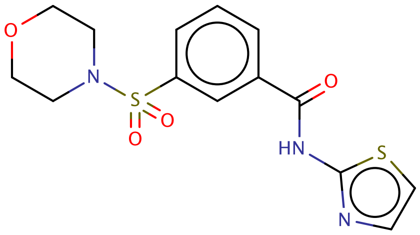	7972860	Inactive	N/A
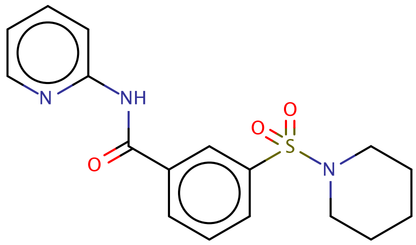	7972957	Inactive	N/A
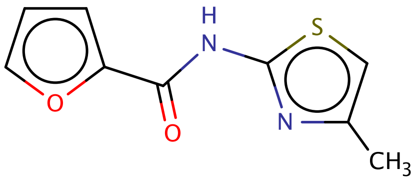	7973018	Inactive	N/A

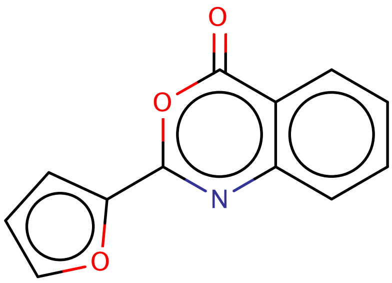	7973318	Active	4.62
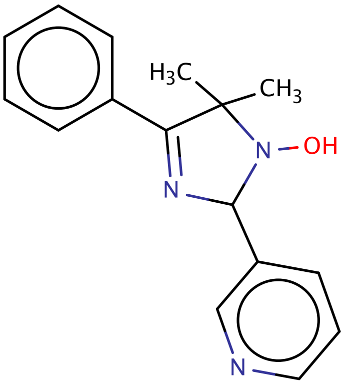	7973389	Inactive	N/A
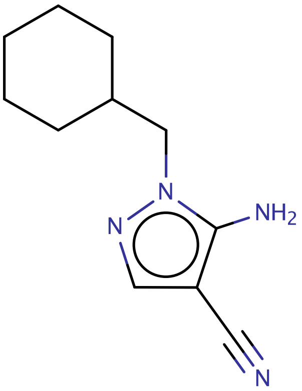	7973713	Inactive	N/A

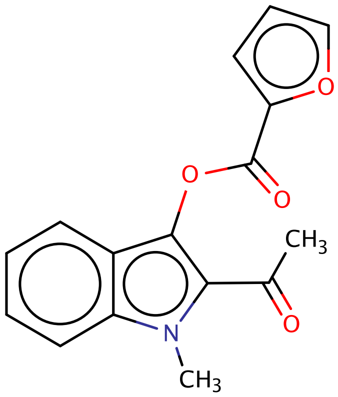	7973891	Active	25.95
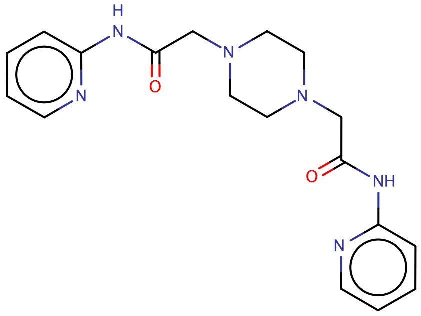	7973916	Inactive	N/A
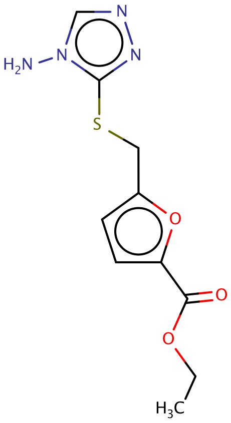	7974410	Inactive	N/A

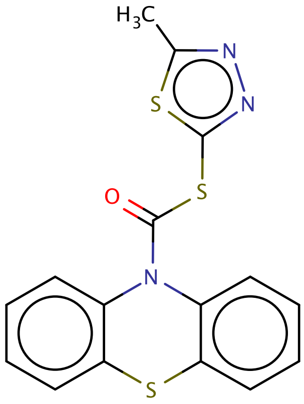	7975174	Inactive	N/A
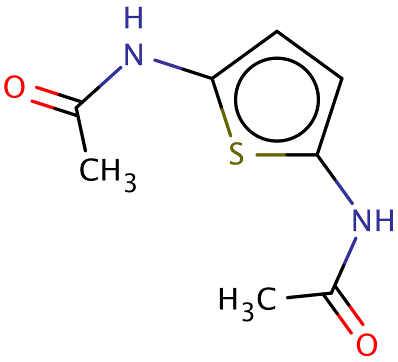	7976940	Inactive	N/A
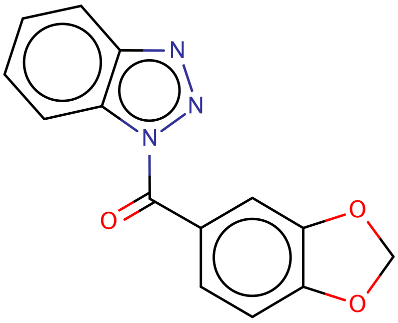	7977382	Active	0.85

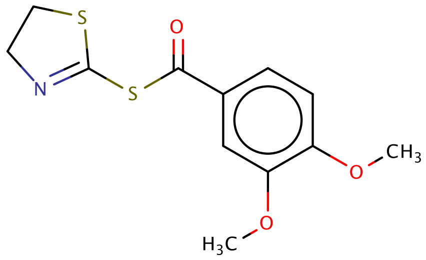	7977978	Inactive	N/A
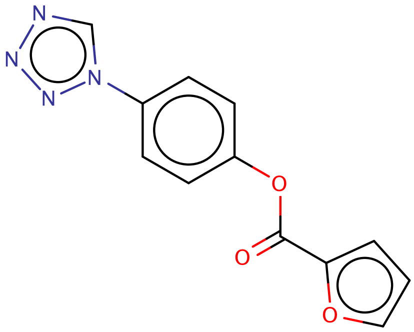	844155	Inactive	N/A
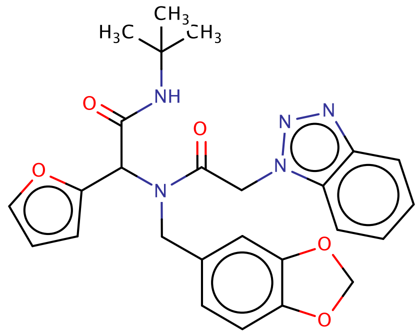	844595	Inactive	N/A

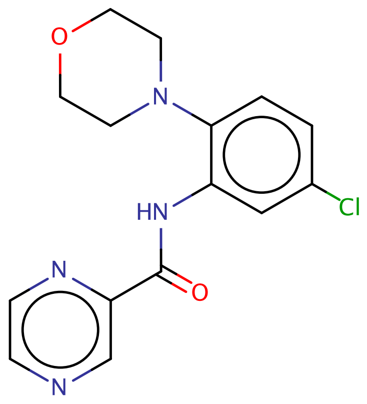	845431	Inactive	N/A
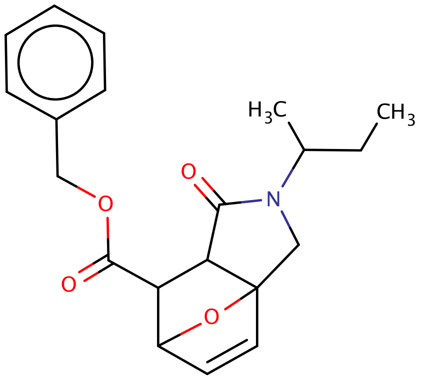	846984	Inactive	N/A
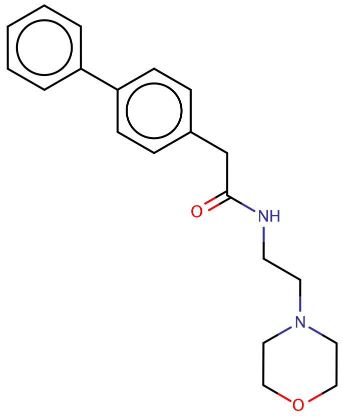	847433	Inactive	N/A

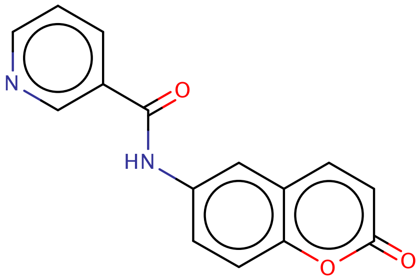	847954	Inactive	N/A
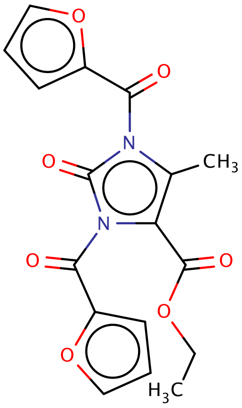	848337	Active	8.54
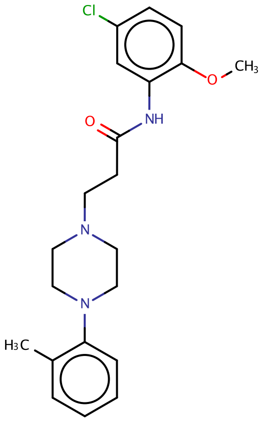	848667	Inactive	N/A

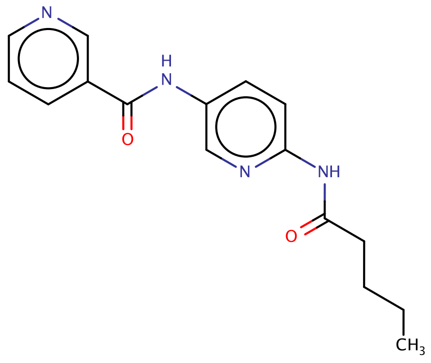	849229	Inactive	N/A
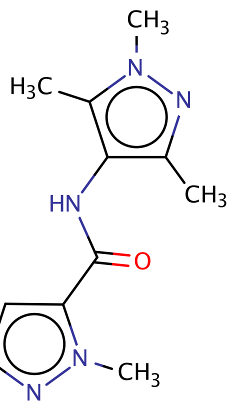	849832	Inactive	N/A
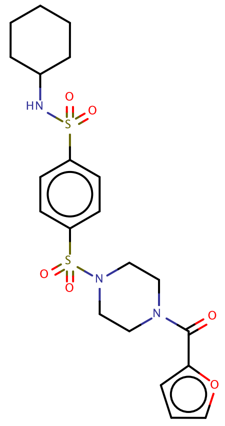	850997	Inactive	N/A

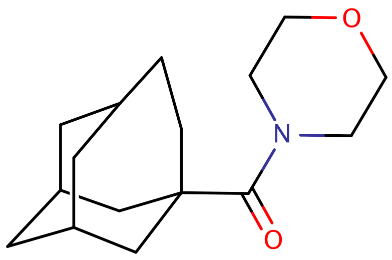	851186	Inactive	N/A
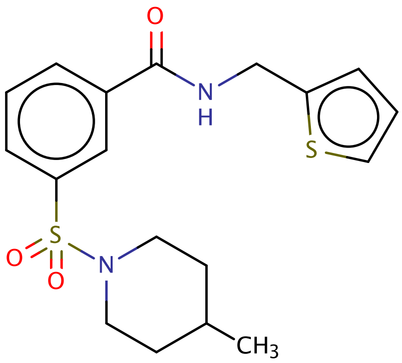	851359	Inactive	N/A
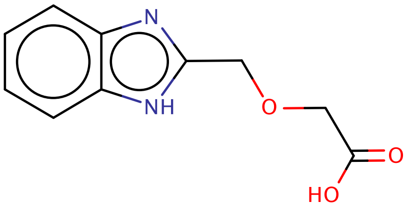	851511	Inactive	N/A

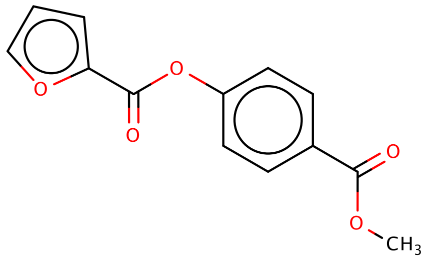	851650	Active	17.07
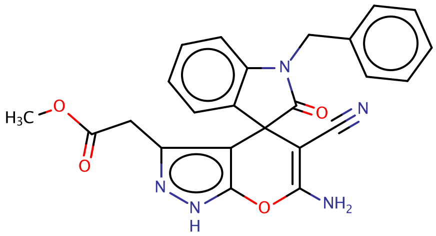	852054	Inactive	N/A
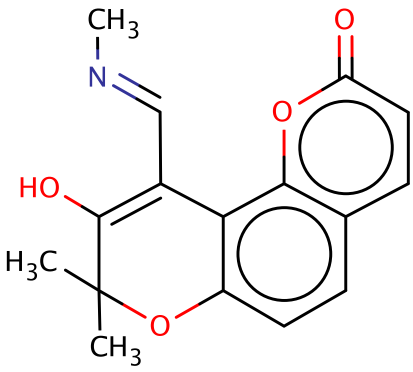	852663	Inactive	N/A

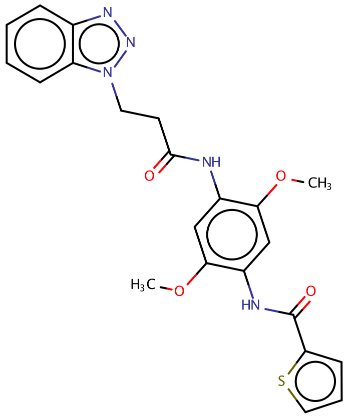	852804	Inactive	N/A
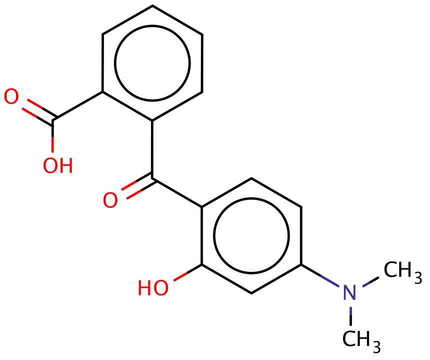	852974	Inactive	N/A
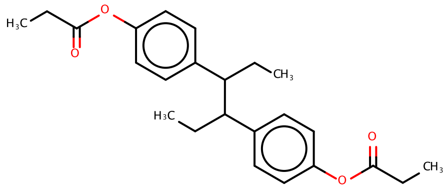	855949	Inactive	N/A

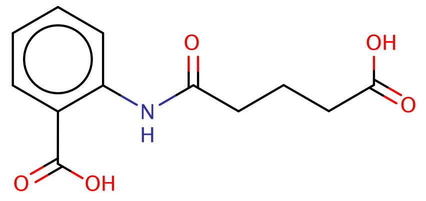	856413	Inactive	N/A
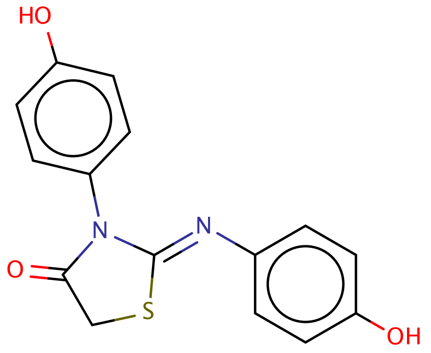	856534	Inactive	N/A
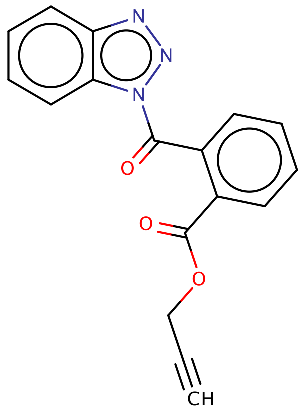	857178	Active	4.26

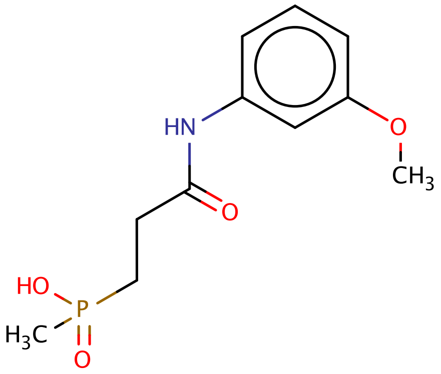	859659	Inactive	N/A
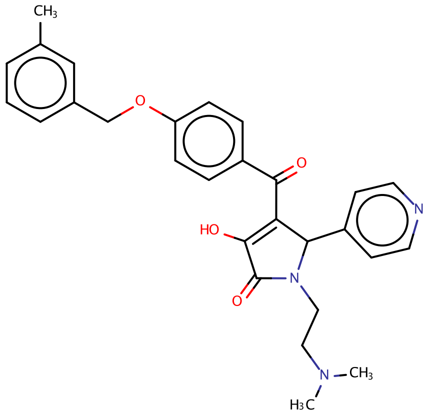	860659	Inactive	N/A
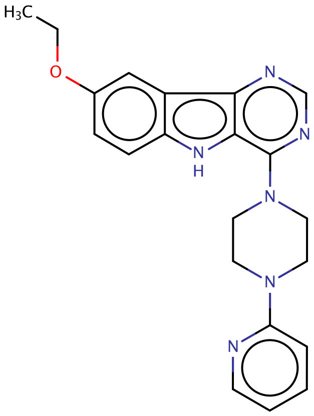	862522	Inactive	N/A

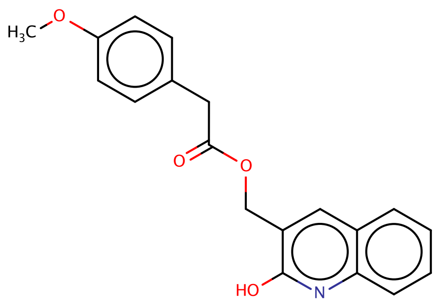	863457	Inactive	N/A
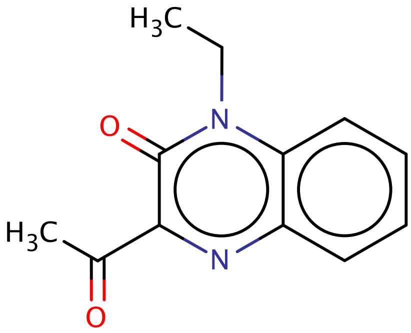	863929	Inactive	N/A
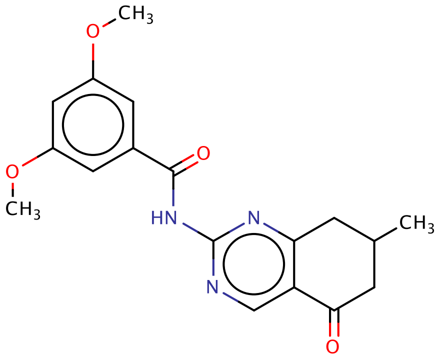	864485	Inactive	N/A

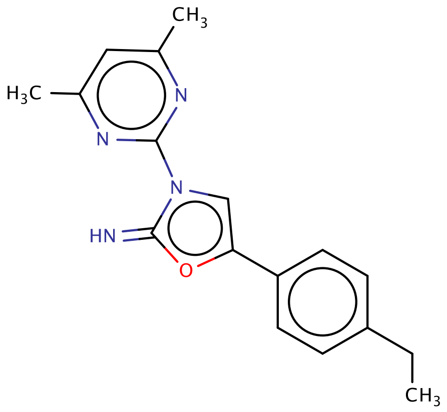	865412	Inactive	N/A
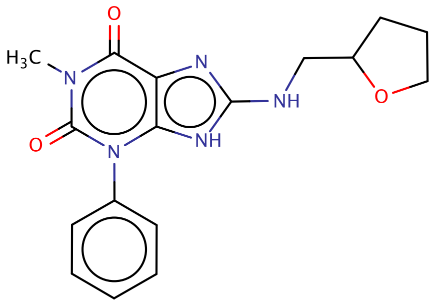	866130	Inactive	N/A
